# Association of genetic variants and survival in patients with acute myeloid leukemia in rural Appalachia

**DOI:** 10.1002/cnr2.1746

**Published:** 2022-11-16

**Authors:** Carl Shultz, Christopher Gates, William Petros, Kelly Ross, Laruen Veltri, Michael Craig, Sijin Wen, Donald A. Primerano, Lori Hazlehurst, James Denvir, Konstantinos Sdrimas

**Affiliations:** ^1^ Osborn Hematopoietic Malignancy and Cellular Therapy Program West Virginia University Department of Hematology/Oncology Morgantown West Virginia USA; ^2^ Department of General Internal Medicine West Virginia University Morgantown West Virginia USA; ^3^ Department of Pharmaceutical Sciences West Virginia University School of Pharmacy Morgantown West Virginia USA; ^4^ Department of Epidemiology and Biostatistics West Virginia University School of Public Health Morgantown West Virginia USA; ^5^ Department of Biomedical Sciences Marshall University, Robert C. Byrd Biotech Center Huntington West Virginia USA

**Keywords:** health disparities, myeloid malignancies, whole exome sequencing

## Abstract

**Background:**

Previous population health studies examining adults with acute myeloid leukemia (AML); however many of these, such as the Cancer Genome Atlas, are derived from databases collected by large urban centers. Due to its unique industry and environmental exposures, we hypothesized the West Virginia Appalachian population may have different mutational trends and clinical outcomes.

**Aims:**

To address the concern of under‐representation of rural minorities in cancer genomic databases, we performed exploratory whole exome sequencing in patients with newly diagnosed AML in rural Appalachia.

**Methods & Results:**

Correlations between genetic variants and clinical outcome variables were examined via retrospective chart review. A total of 26 patients were identified and whole exome sequencing was performed. Median age was 68 years old. Twenty‐one patients had de novo AML (84%). As per European LeukemiaNet (ELN) criteria, 8 patients were favorable (32%), 12 were intermediate (48%), and 5 were adverse risk (20%). Eight patients proceeded to transplant. The median progression‐free survival and overall survival were 16.5 months and 26.6 months, respectively. We noted an increased tumor mutation burden and a higher frequency of specific known driver mutations when compared to The Cancer Genome Atlas database; we also found novel mutations in *MUC3A*, *MUC5AC*, *HCAR3*, *ORT2B*, and *PABPC*. Survival outcomes were slightly lower than national average and *BCOR* mutation correlated with inferior outcomes.

**Conclusion:**

Our findings provide novel insight into detrimental mutations in AML in a rural, underrepresented population. We discovered several novel mutations and higher frequency of some known driver mutations, which will help us identify therapeutic targets to improve patient outcomes.

## 1 INTRODUCTION

Acute myeloid leukemia (AML) is the most common type of acute leukemia in adults. In the United States and Europe, the incidence is reported as 3–5 cases per 100 000 population with a median age at diagnosis of 65 years. Incidence increases with age from approximately 2 cases per 100 000 population for those under 65 years to 20 cases per 100 000 population for those over 65 years. Additionally, the incidence varies among different races with non‐Hispanic Whites having the highest incidence—4 cases per 100 000 population. The cause of AML is not clear; however, it has been associated with environmental factors (e.g., chemical exposure, radiation, chemotherapy, and tobacco), genetic abnormalities (e.g., trisomy 21, Fanconi anemia, familial mutations of *CEBPA*, *DDX41*, and *RUNX1*), and evolution from clonal hematopoietic disease states (e.g., myelodysplastic syndrome, myeloproliferative neoplasm, and aplastic anemia). Despite increased knowledge of the disease biology and availability of cytogenetic analyses and molecular testing, the 5‐year overall survival (OS) is a disappointing 28.3%.^1^, ^2^, ^3^

While there have been previous population health studies examining children and adults with leukemia, many of these studies are derived from databases collected by large urban centers.^4^, ^5^ The 2017 European LeukemiaNet (ELN) criteria are widely used to stratify patients into three prognostic groups: favorable, intermediate, and adverse. Since then, there have been many gene mutations identified which are not currently characterized in this model that may have a significant impact on prognosis, such as *NRAS*/*KRAS* or *BCOR* mutations. Recent advances using targeted therapy for patients who are not candidates for intensive induction chemotherapy and who have relapsed or refractory disease have shown improved response rates.^6^

To address the concern of under‐representation of rural Appalachian population in cancer genomic databases, we performed exploratory whole exome sequencing in patients with newly diagnosed AML. We analyzed patients with newly diagnosed AML, whose bone marrow and buccal samples were obtained as part of the “Collection of Human Blood and Tissue for Research Purposes in the West Virginia University Tissue Bank” from 2015 to 2017. As the only stem cell transplantation center in the state, West Virginia University Hospital serves many regional patients with hematologic malignancies and therefore, is uniquely situated to capture most of these patients from the state and surrounding area. As per the Appalachian Regional Commission, West Virginia is the only state that is considered located entirely within Appalachia. In addition to identifying mutational trends, we aimed to assess clinical outcomes.

## 2 METHODS

Patients with a new diagnosis of AML were consented for genetic analysis under an approved IRB protocol at West Virginia University [“Collection of Human Blood and Tissue for Research Purposes in the West Virginia University Biorepository” (IRB #1310105737).] Inclusion criteria included age greater than 18 years old, new diagnosis of AML, and signed informed consent at time of enrollment. Exclusion criteria included prior history of AML (refractory or relapsed) and history of allogenic stem cell transplant. Genomic DNA was extracted from patient bone marrow and buccal swab tissue using the Qiagen (Germantown, MD) DNeasy Blood and Tissue Protocol method. De‐identified DNA samples were shipped to the Marshall University (MU) Genomics Core by next day delivery.

In order to identify genetic variants in known and potential cancer‐associated genes, we performed whole exome sequencing on bone marrow and buccal tissue from AML patients. Variants that were present in buccal tissue were considered to be germline and were removed from consideration given the scope of this study. Whole exome sequencing libraries were prepared from patient genomic DNA using Agilent (Santa Clara, CA) SureSelect Human Whole Exome technology. Whole exome libraries were sequenced on an Illumina HiSeq1500 sequencer in the MU Genomics Core in a series of paired end runs between 2015 through 2017. Reads were aligned to the human reference genome GRCh38 using BWA, and base calls were recalibrated using the Genome Analysis Toolkit (GATK version 3.8.0, BROAD Institute, Cambridge, MA) BaseRecalibrator tool, with SNPs from dbSnp version 146 and indels from the Mills and 1000 genomes gold standard as references. Somatic mutations were identified using the GATK mutect2 tool, and filtered using the GATK tools CalculateContamination and FilterMutectCalls, all with default parameters. Downstream analysis of our cohort and the TCGA AML cohort was performed using the *maftools* Bioconductor package using prespecified parameters.^7^ Single‐nucleotide variation mutation annotation format (MAF) files were downloaded from the Genomic Data Commons (GDC) data portal from AML patients with clinical data, whole exome sequencing, and masked somatic mutations MAF files available (*n* = 143). All de‐identified variant data are stored on a password‐protected server maintained by the Marshall University Bioinformatics Core Facility housed in the Drinko Data Center at the Marshall University.

We performed a retrospective chart review to examine correlations between identified genetic variants and clinical outcome variables. We evaluated associations between genetic data and progression free survival (PFS) and overall survival (OS). The Kaplan–Meier method was used to estimate the survival curves of time‐to‐event data. The log‐rank test was used to compare survival distributions between groups.

## 3 RESULTS

A total of 26 patients were identified who met the inclusion criteria for newly diagnosed AML criteria in this study. Table 1 provides an overview of patient characteristics. The median age of the entire cohort was 68 years. Women represented 10 of the patients (38.4%). Median Eastern Cooperative Oncology Group (ECOG) Performance Status assessed near the time of sample collection was 2. Two patients had other malignancies present including breast and kidney cancer (7.6%). Twenty‐two patients had de novo AML (84.6%), three patients had preceding Myelodysplastic Syndrome (MDS, 11.6%), and one patient had therapy‐related AML (3.8%). The median white blood cell count at diagnosis was 44.65 × 10^9^/L (range 0.4–199 × 10^9^/L). The median blast count at diagnosis was 49% (range: 0–96%). When risk was stratified per European Leukemia Network (ELN) criteria, eight patients were favorable (30.7%), 12 were intermediate (50%), and five were adverse risk (19.2%). Remission status was determined by a day 28–30 bone marrow analysis using remission criteria as outlined by the International Working Group.^8^ Eighteen patients achieved Complete Remission (CR, 69.2%), five patients achieved Partial Remission (PR, 19.2%), two patients died during induction (7.6%), and one patient had no response (3.8%). The median progression free survival and median overall survival were 16.5 months and 26.6 months, respectively.

**TABLE 1:** Patient characteristics

Characteristic	*n* = 26 (%)
Female gender	10 (38.4%)
Age in years, median (range)	68 (20–85)
ECOG, median (range)	2 (0–4)
Other malignancies present	2 (7.6%)
HCT‐CI, median (range)	2 (0–6)
*Type of AML*	
De‐novo	22 (84.6%)
MDS‐related	3 (11.6%)
Therapy‐related	1 (3.8%)
WBC at diagnosis, median (range x 10^9/L)	44.65 (0.4–199)
Blast count at diagnosis in PB, median (range)	49 (0–96%)
*ELN 2017 risk category*	
Favorable	8 (30.7%)
Intermediate	13 (50%)
Adverse	5 (19.2%)
*Day 28 bone marrow remission status*	
CR/CRi	18 (69.2%)
PR	5 (19.2%)
NR	1 (3.8%)
Death during induction	2 (7.6%)
Subsequent chemotherapy after induction	14 (53.8%)
Allogeneic HSCT	8 (33.3%)
MUD	4 (50%)
Haploidentical	1 (12.5%)
Cord	3 (37.5%)
*Disease Risk Index Score*	
Low	1 (12.5%)
Medium	3 (37.5%)
High	4 (50.0%)
Very high	0 (0%)
Median progression free survival	16.5 months (393 days)
Median overall survival	26.6 months (503 days)

Among those who were transplant eligible, four were intermediate risk, two were adverse, and two were favorable. The CIMBTR risk was similar: four were high, three were medium and one was low risk. Donor source varied: four were matched‐unrelated donors (50%), three were dual umbilical cord (37.5%), and one was haploidentical (12.5%). Of these eight patients, seven were a disease risk index (DRI) of medium or higher. The median overall survival for this group was 51.3 months.

In order to identify genes of interest in our cohort, we ranked genes by the number of patients that had at least one coding or splice region variant in a given gene which was not present in that patient's buccal (germline) sample. We found variants mapping to one gene that were present in multiple patients. Six of our 26 AML patients had mutations in the *NPM1* gene, followed by *DNMT3A* (5 patients), *TET2* (4 patients one of which had two mutations) and *IDH1* (4 patients). Nine genes had coding/splice variants which were present in three AML patients (Supplemental Table 1). Many, but not all, of the genes identified in this ranking have been identified as AML drivers in larger studies.^4^

We also searched for mutations in our dataset using the Mayo OncoHeme Next‐Generation Sequencing (NGS) 42‐gene panel: *ANKRD26*, *ASXL1*, *BCOR*, *CALR*, *CBL*, *CEBPA*, *CSF3R*, *DDX41*, *DNMT3A*, *ELANE*, *ETNK1*, *ETV6*, *EZH2*, *FLT3*, *GATA1*, *GATA2*, *IDH1*, *IDH2*, *JAK2*, *KDM6A*, *KIT*, *KRAS*, *MPL*, *NPM1*, *NRAS*, *PHF6*, *PTPN11*, *RAD21*, *RUNX1*, *SETBP1*, *SH2B3*, *SF3B1*, *SRP72*, *SMC3*, *SRSF2*, *STAG2*, *TERT*, *TET2*, *TP53*, *U2AF1*, *WT1*, and *ZRSR2*. Oncoplots comparing the percentage of patients with mutations in each gene along with the percentages obtained from TCGA database can be found in the supplementary section (Supplemental Figure1). Our patient population had a higher frequency of *BCOR* (8%), and *TET2* (12%) coding mutations when compared to previous large national data sets.^4^, ^5^

**FIGURE 1:**
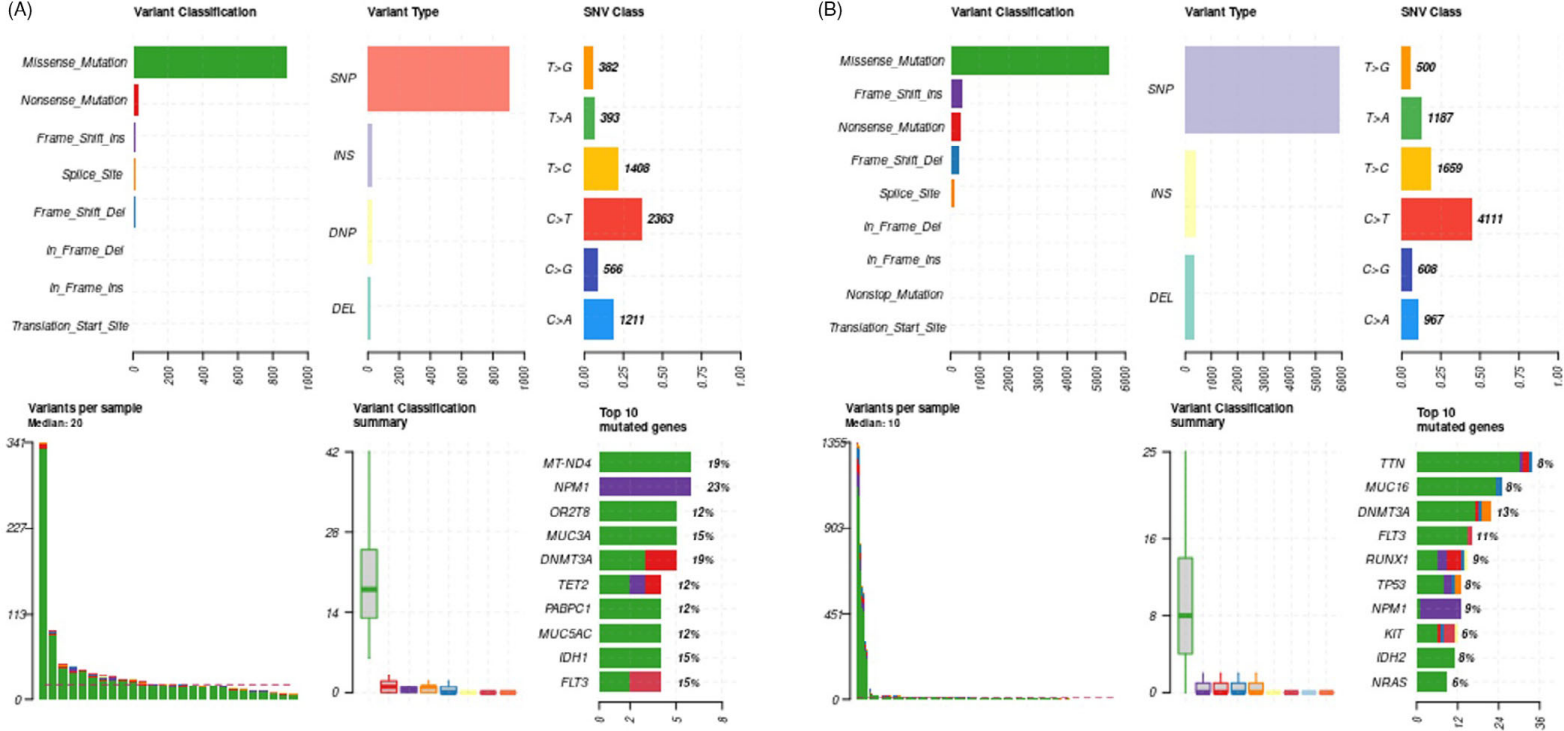
Characterization of Mutations. Panel A shows the 10 most common gene mutations in our cohort. Each bar represents a distinct driver lesion with associated variant classification, variant type, single‐nucleotide variant (SNV), and number of variants per sample. Panel B shows the 10 most common gene mutations as per The Cancer Genome Atlas Research Network (TCGA). Each bar represents a distinct driver lesion with associated variant classification, variant type, single‐nucleotide variant (SNV), and number of variants per sample.

**FIGURE 2:**
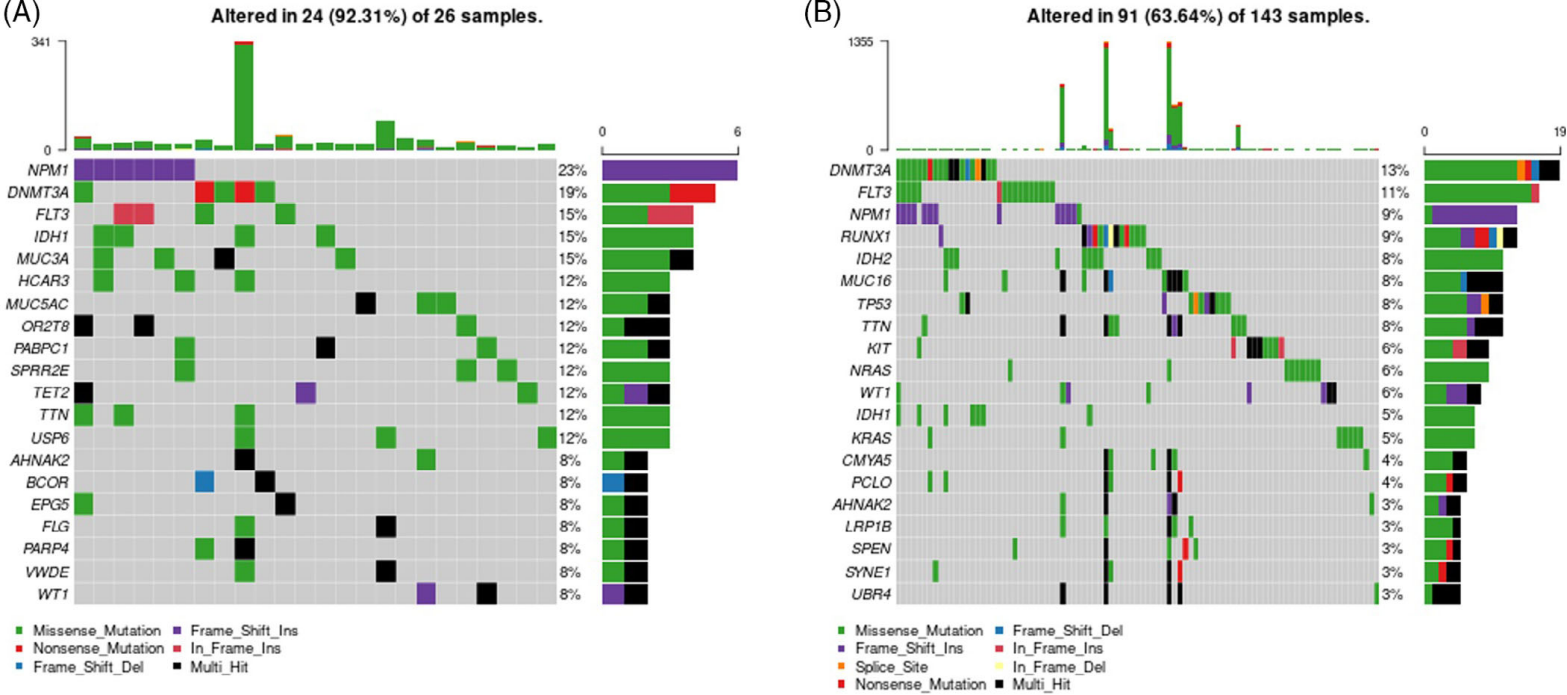
Mutational Landscape. Panel A shows oncoplot of the 20 most commonly mutated genes of interest in our patient cohort compared to the TCGA database, Panel B.

The 10 most common gene mutations with each bar representing a distinct driver and associated variant classification, variant type, single‐nucleotide variant (SNV), and number of variants per sample as compared to TCGA database are shown in Figure 1. Median variants per sample were 20 in our data set compared to 10 in the TCGA database. While some genes overlapped as most common including *NPM1*, *DNMT3A*, and *FLT3*, some were mutated more frequently in our patients including *TET2* and *IDH1*. Additionally, two mutations in *MUC3A* (15%) and *MUC5AC* (12%) were identified in our cohort that were not present in TCGA database; conversely, *MUC16* (8%) was observed in TCGA but not the WV cohort. Figure 2 demonstrates oncoplots of the most commonly mutated genes in AML as per whole exome sequencing in our patient population as compared to TCGA database.

**FIGURE 3:**
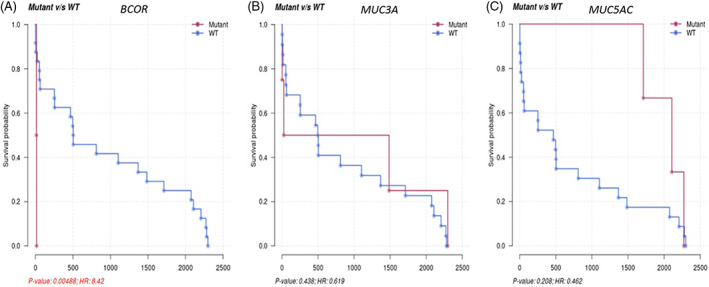
Molecular Subclassification and Overall Survival. Panel A shows Kaplan–Meier curves for overall survival among patients with *BCOR* mutation (2 patients). Panel B shows Kaplan–Meier curves for overall survival among patients with *MUC3A* mutation (4 patients). Panel C shows Kaplan–Meier curves for overall survival among patients with *MUC5AC* mutation (3 patients).

**FIGURE 4:**
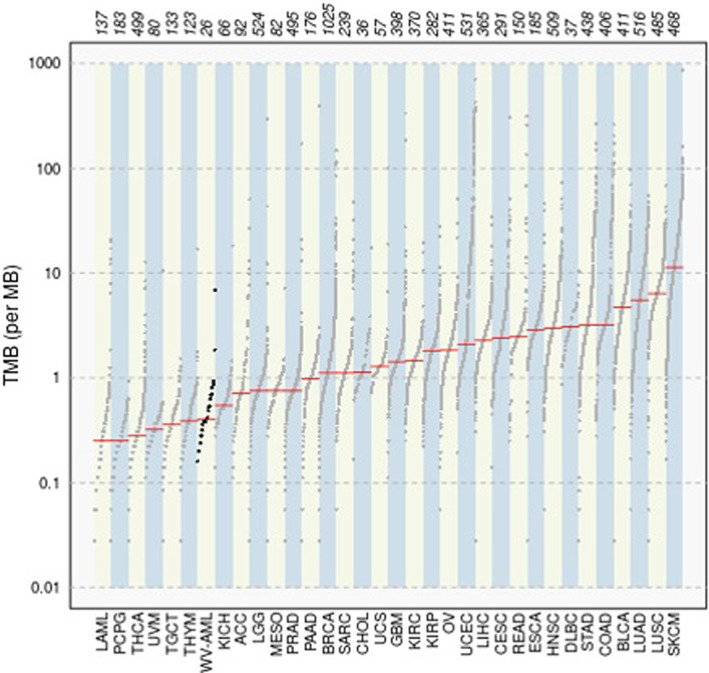
Mutational Load. Panel A shows total mutational burden (TMB), the number of non‐synonymous, somatic mutations identified per megabase (Mb) of the genome coding area of DNA, on y‐axis with the corresponding TCGA cohorts of all cancer subtypes on x‐axis. Highlighted is the TMB for patients in West Virginia (WV‐AML) as compared to the TCGA AML cohort (LAML).

**FIGURE 5:**
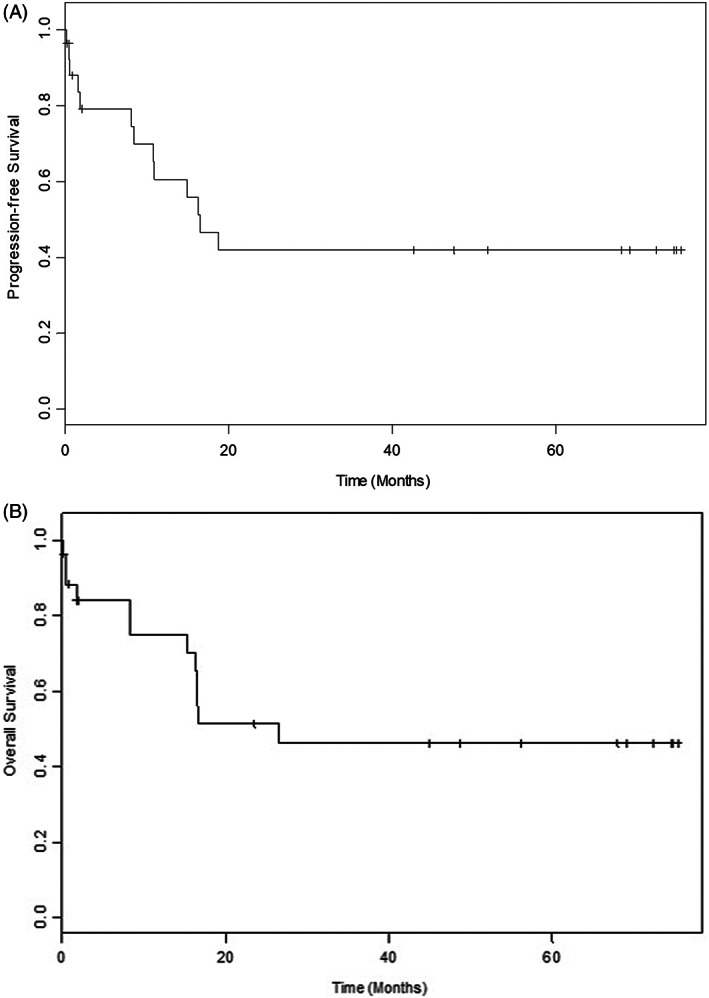
a. Kaplan–Meier curve demonstrating progression‐free survival (PFS) in months (median = 16.5). b. Kaplan–Meier curve demonstrating overall survival (OS) in months (median = 26.6).

Figure 3 shows Kaplan–Meier curves demonstrating overall survival as per molecular subclassification based on three genes: *BCOR*, *MUC3A*, and *MUC5AC*. While *BCOR* was noted in both datasets, it was more frequently mutated in the WV cohort and the latter two mutations were not seen in the TCGA database. *BCOR* mutation correlated with poor outcomes in our study (p‐value = 0.00488; HR = 8.42). On the other hand, mutations in the *MUC3A* and *MUC5AC* gene correlated with favorable outcomes (p‐value: 0.438; p‐value: 0.208). Additionally, our patient population (WV‐AML) had a higher mutation load as compared to TCGA (i.e., LAML; see Figure 4). Median tumor mutation burden per MB was 0.4 and 0.25, respectively. Finally, Figure 5 shows Kaplan–Meier curve demonstration progression free survival of 16.5 months for entire patient cohort, while Figure 5b shows an overall survival of 26.6 months.

## 4 DISCUSSION

Using whole exome sequencing, we analyzed the genomic landscape and clinical outcomes of patients with de novo AML in West Virginia, a rural Appalachian population. Median progression free survival and overall survival outcomes were 16.5 and 26.6 months, respectively. The estimated 5‐year overall survival was 23.1%, which was lower than the national survival rate of 28.3%. Of our 26 patients, 8 underwent allogeneic transplant. We noted a similar mutational trend nationally, except for a higher frequency of some mutations including: *MUC3A*, *MUC5AC*, *BCOR*, *HCAR3*, *ORT2B*, and *PABPC1*. Finally, our patient population had a higher tumor mutation burden when compared to prior AML data sets.

The mucin (MUC) family is a group of highly glycosylated proteins that are abundantly expressed in mammalian epithelial cells and contribute to the formation of mucus barriers. Some MUCs are aberrantly expressed in cancer cells and are involved in cancer development and progression. With their unique biological and structural features, MUC proteins have been considered promising therapeutic targets and also biomarkers for human cancer.^9^ Interestingly, mutations in two specific MUC genes were identified in our patient cohort: *MUC3A* and *MUC5AC*. Four of our patients had *MUC3A* mutations and three had *MUC5AC* mutations. An association with *MUC3A* expression and poor prognosis has been associated with gastrointestinal malignancies (e.g., gastric, pancreatic), breast, and renal cancers.^10^ However, little is known about the functional role of *MUC3A* in hematologic malignancies including AML. Interestingly, neither *MUC3A* nor *MUC5AC* were identified in the TCGA database.

The BCL6 co‐repressor (*BCOR*) gene, located on chromosome Xp114, consists of 15 exons.^11^ Its product is a nucleoprotein known to play an essential role in normal hematopoiesis as a transcription regulatory factor known to inhibit myeloid cell proliferation and differentiation and suppressant action on the apoptosis‐related protein BCL6. Likewise mutations in BCOR skew hematopoiesis toward the myeloid lineage using mouse models of hematopoiesis.^12^ Grossmann et al. reported that adult patients with de novo AML with normal karyotype had a *BCOR* mutation frequency in approximately 4% of cases (10 out of 262 cases). Interestingly, they also showed a tendency to infrequently co‐occur with *FLT3‐ITD* and *NPM1* and showed worse prognosis than other patients with AML and normal karyotype.^13^ Tereda et al. found that 7% of patients (28 out of 377) with de novo AML had a *BCOR* or *BCORL1* mutation. Among cases aged 65 years or younger, *FLT3‐ITD* negative and intermediate cytogenetic prognosis group, *BCOR* was associated with a lower 5‐year overall survival and relapse free survival. Additionally, multivariate analysis demonstrated that *BCOR* mutations were an independent prognostic factor.^14^ Of our two patients with *BCOR* mutations, both had a co‐existing *FLT3‐ITD* mutation, one of which also had a *NPM1* mutation. One patient had secondary AML, while the other had de novo disease. One was female and the average age was 73 years old. Both patients were intermediate risk and both died during induction. The median overall survival was 15 days. *BCOR* mutation correlated with poor outcomes in our study (*p*‐value = 0.00488; HR = 8.42); however, a larger cohort is needed to validate these findings. Currently, there is no drug regimen that is clinically directed by *BCOR* mutations and further pre‐clinical studies are required to better understand the actionable vulnerabilities in cells harboring *BCOR* mutations.

The G‐protein‐coupled receptors (GPCRs) family constitute the largest and most diverse receptor family in the human genome. Of these, hydroxycarboxylic acid receptors (*HCAR*) and olfactory receptor family 2 subfamily T member 8 (*OR2T8*) are two novel ones discovered in our patients. *HCAR* is a metabolite‐sensing GPCR and has shown a role in cancer pathogenesis.^15^ Specifically, it has shown higher expression in colorectal cancer and demonstrated better overall survival outcomes.^16^ Little is known about its significance in AML. *OR2T8* is an olfactory membrane receptor protein. Olfactory receptors share a 7‐transmembran domain structure with many neurotransmitter and hormone receptors. They are responsible for the recognition and the G protein‐mediated transduction of odorant signals.^17^ Little is known about its pathogenesis in AML.

Poly(A)‐binding protein cytoplasmic 1 (*PABPC1*) gene encodes for a poly(A) protein, which shuttles between the nucleus and cytoplasm and binds to the 3′ poly(A) tail of messenger RNA (mRNA). It plays a pivotal role in translation initiation by combining with eukaryotic initiation factor 4G (eIF4G).^18^ Recently, a small peptide produced from non‐coding RNA known as APPLE (A Peptide Located in the Endoplasmic Reticulum) showed enhanced translation via the PABPC1‐eIF4G complex. Its oncogenic potential was noted in primary cells derived from AML.

Some of our patients carried mutations in two or more known drivers. One patient had missense mutations in *TET2*, *BCOR* and *KRAS* for example. The total number of missense mutations present in our patients ranged from 9 to 371. In addition to known driver mutations in *DNMT3A* and *IDH1*, one patient also had missense tumor specific mutations in the *FANCA* and *FANCM* genes. Given the absence of these mutations in normal tissue and the role of the FA complex in DNA interstrand crosslink repair and replication rescue,^19^ the high number/frequency of mutations can be attributed to disruption of DNA repair in tumor tissue. Additional consideration is whether patients harboring tumor specific FA mutations would be candidates for a synthetic lethality strategy utilizing a PARP inhibitor. To address these questions matching ex‐vivo drug sensitivity with OMIC approaches may lead to strategies to individualize the treatment for AML.^20^

Our study sought to assess gene mutation variants, mutational burden, and survival outcomes in the context of a population living in a state with a high tobacco‐smoking rate and industrial exposures including coal mining and chemical manufacturing. Given the retrospective nature of this study, it was not possible to consistently document potential environmental exposures for individual patients. The largest known exposure was tobacco use, having seven patients with documented use. Our study had several limitations including small sample size, retrospective nature, and single, regional medical center. Furthermore, the novel findings of this preliminary study require validation in a larger cohort with different time scales. While germline variant data was not pursued given the focus of this study, this is an area of rapidly expanding knowledge—as evidenced by the World Health Organization, National Comprehensive Cancer Network, and ELN further recognizing the germline predisposition to MDS/AML—and one of future investigation particularly in our patient population. The findings provide further assessment of predisposing detrimental mutations in AML in the Appalachian region and associated survival outcomes.

## AUTHOR CONTRIBUTIONS

**Carl Shultz:** Formal analysis (equal); investigation (equal); visualization (equal); writing – original draft (equal); writing – review and editing (equal). **Christopher Gates:** Investigation (equal); writing – review and editing (equal). **William Petros:** Conceptualization (equal); funding acquisition (equal); supervision (equal); writing – review and editing (equal). **Kelly Ross:** Investigation (equal). **Lauren Veltri:** Formal analysis (equal); investigation (equal). **Michael Craig:** Investigation (equal). **Sijin Wen:** Formal analysis (equal); visualization (equal); writing – review and editing (equal). **Donald A. Primerano:** Formal analysis (equal); supervision (equal); writing – original draft (equal); writing – review and editing (equal). **Lori Hazlehurst:** Conceptualization (equal); formal analysis (equal); funding acquisition (equal); methodology (equal); supervision (equal); writing – original draft (equal); writing – review and editing (equal). **James Denvir:** Formal analysis (equal); supervision (equal); writing – original draft (equal); writing – review and editing (equal). **Konstantinos Sdrimas:** Conceptualization (equal); formal analysis (equal); methodology (equal); supervision (equal); visualization (equal); writing – original draft (equal); writing – review and editing (equal).

## CONFLICT OF INTEREST

The authors have no conflicts interest.

## ETHICAL STATEMENT

This study received IRB approval from West Virginia University and patient consent was obtained.

## Supporting information


**Appendix S1.** Supporting Information

**Figure S1:** Mutational Landscape. Panel A shows oncoplot of mutated genes included in Mayo OncoHeme gene list compared to those of Panel B, the TCGA database.

**Supplemental Table 1:** List of mutated genes with variant classification and variant type for each patient.”

## Data Availability

The datasets generated during and/or analyzed during the current study are available from the corresponding author on reasonable request.
