# Supplementary material for: Association of genetic variants and survival in patients with acute myeloid leukemia in rural Appalachia
Source: Cancer Rep (Hoboken). 2022 Nov 16;6(3):e1746. doi: 10.1002/cnr2.1746 (PMC10026309; doi:10.1002/cnr2.1746)
Supplement: Supplementary file 1 — Appendix S1. Supporting Information [file CNR2-6-e1746-s001.pdf]

All Patients

Patient ID	Hugo Symbol	Chromosome	Start Position	Variant Classification	Variant Type	Reference Allele	Tumor Allele
1	AKR1C1	10	4972686	Silent	SNP	A	G
1	ANK2	4	113356595	Silent	SNP	G	A
1	DGKD	2	233441940	Missense_Mutation	SNP	A	G
1	EXOC6B	2	72480710	Missense_Mutation	SNP	G	A
1	FAM131C	1	16058503	Missense_Mutation	DNP	GG	CA
1	GEN1	2	17761589	Splice_Region	SNP	A	G
1	IGSF3	1	116603941	Missense_Mutation	SNP	G	A
1	IRAG1	11	10626155	Silent	SNP	G	A
1	KIT	4	54733155	Missense_Mutation	SNP	A	T
1	KLHL20	1	173775681	Missense_Mutation	SNP	A	G
1	KLKB1	4	186238320	Missense_Mutation	SNP	G	A
1	NRAS	1	114716126	Missense_Mutation	SNP	C	T
1	PITPNM1	11	67499962	Missense_Mutation	SNP	C	T
1	PTCHD4	6	48009046	Silent	SNP	C	T
1	RPL3	22	39314206	Splice_Region	SNP	A	G
1	RRH	4	109844157	Missense_Mutation	SNP	T	C
1	SCARA5	8	27987610	Missense_Mutation	SNP	C	A
1	SHOX2	3	158105051	Splice_Site	DEL	AACACA	-
1	SON	21	33559643	Missense_Mutation	SNP	C	A
1	SPRR2E	1	153093591	Missense_Mutation	SNP	C	T
1	SPRR2E	1	153093596	Silent	SNP	T	C
2	CBL	11	119278169	Missense_Mutation	SNP	C	A
2	CDV3	3	133586635	Missense_Mutation	SNP	G	C
2	CELA3A	1	22005699	Missense_Mutation	SNP	A	G
2	CELA3A	1	22005703	Missense_Mutation	SNP	T	G
2	CLRN1	3	150941728	Missense_Mutation	SNP	C	T
2	EPHA2	1	16133250	Silent	SNP	G	A
2	EZH2	7	148828739	Splice_Site	SNP	C	T
2	FLRT3	20	14325873	Missense_Mutation	SNP	A	G
2	HCAR3	12	122715980	Missense_Mutation	SNP	T	C
2	INTS5	11	62649459	Silent	SNP	C	T
2	IRF2BPL	14	77027302	In_Frame_Del	DEL	GCA	-
2	LGALS16	19	39658654	Missense_Mutation	SNP	G	C
2	NLRP5	19	56026928	Missense_Mutation	SNP	C	T
2	NPM1	5	171410539	Frame_Shift_Ins	INS	-	TCTG

2	NR4A3	9	99828760	Missense_Mutation	SNP	A	G
2	OCA2	15	27755455	Missense_Mutation	SNP	A	G
2	OR52N2	11	5821080	Missense_Mutation	SNP	T	G
2	PABPC1	8	100705604	Missense_Mutation	SNP	G	C
2	PCDH15	10	53938856	Missense_Mutation	SNP	G	A
2	PCDH15	10	53866863	Splice_Region	SNP	C	T
2	PCDH18	4	137529790	Missense_Mutation	SNP	C	A
2	PTCH1	9	95467108	Splice_Region	SNP	C	T
2	SPRR2E	1	153093591	Missense_Mutation	SNP	C	T
2	SPRR2E	1	153093596	Silent	SNP	T	C
3	ADCY2	5	7766780	Missense_Mutation	SNP	C	T
3	CAV3	3	8745810	Silent	SNP	C	T
3	DLL3	19	39505307	Missense_Mutation	SNP	G	A
3	FLNC	7	128843841	Missense_Mutation	SNP	A	C
3	FREM1	9	14775781	Splice_Region	SNP	A	G
3	GFRA3	5	138257870	Missense_Mutation	SNP	G	A
3	GRHL3	1	24342935	Silent	SNP	G	A
3	H1-1	6	26017366	Missense_Mutation	SNP	C	T
3	IRAG1	11	10594172	Missense_Mutation	SNP	G	A
3	MCAM	11	119312782	Missense_Mutation	SNP	C	A
3	MT-ND6	MT	14470	Silent	SNP	T	A
3	OR8B3	11	124397252	Missense_Mutation	SNP	C	T
3	PER1	17	8147388	Splice_Region	SNP	C	A
3	PLVAP	19	17360807	Missense_Mutation	SNP	C	T
3	RPL36	19	5690576	Silent	SNP	G	A
3	SCRN2	17	47838797	Missense_Mutation	SNP	G	A
3	SLC12A2	5	128084626	Silent	SNP	C	A
3	SLC30A2	1	26045874	Frame_Shift_Ins	INS	-	GG
3	SYT4	18	43271763	Missense_Mutation	SNP	C	T
4	ADAMTSL3	15	83773552	Silent	SNP	C	T
4	ANKRD36C	2	95939004	Nonsense_Mutation	SNP	G	A
4	ARRDC4	15	97969875	Splice_Region	SNP	C	T
4	ATXN7L1	7	105665128	Silent	SNP	T	C
4	CAP2	6	17507270	Silent	SNP	G	A
4	CCDC168	13	102744419	Missense_Mutation	SNP	C	T
4	CEP350	1	179987270	Missense_Mutation	SNP	C	G
4	CLIP1	12	122355190	Silent	SNP	C	T

4	CNTN3		3	74285378	Silent	SNP	C	T
4	CSPG4		15	75689744	Missense_Mutation	SNP	C	T
4	DDX53	X		23000533	Missense_Mutation	SNP	C	A
4	DELEC1		9	115401310	Splice_Region	SNP	G	T
4	FAM171B		2	186694193	Missense_Mutation	SNP	G	A
4	FBXO3		11	33774409	Missense_Mutation	SNP	T	C
4	GOLGA6L6		15	20534793	Missense_Mutation	SNP	C	G
4	IDH1		2	208248388	Missense_Mutation	SNP	C	T
4	LRIG2		1	113116282	Splice_Region	SNP	T	C
4	MUC17		7	101041742	Silent	SNP	C	T
4	MYO18B		22	26027404	Missense_Mutation	SNP	C	T
4	PABPC1		8	100709477	Missense_Mutation	SNP	G	T
4	PABPC1		8	100709481	Missense_Mutation	SNP	T	A
4	PCDH15		10	54066775	Silent	SNP	G	T
4	PCDHGB1		5	141351683	Missense_Mutation	SNP	C	T
4	PLAC9		10	80144297	Missense_Mutation	SNP	G	C
4	PNPLA2		11	824669	Missense_Mutation	SNP	G	C
4	PROZ		13	113171852	Missense_Mutation	SNP	G	A
4	PTPN4		2	119945160	Missense_Mutation	SNP	C	G
4	PXN		12	120213982	Nonsense_Mutation	SNP	C	A
4	RHPN1		8	143382559	Missense_Mutation	SNP	G	T
4	SMARCA4		19	11033470	Missense_Mutation	SNP	C	T
4	SMC1A	X		53414845	Missense_Mutation	SNP	G	T
4	TMEM87B		2	112091789	Splice_Region	SNP	C	T
4	U2AF2		19	55660514	Splice_Site	SNP	A	C
4	UBR4		1	19152308	Splice_Region	SNP	C	A
4	UGT2B28		4	69294559	Missense_Mutation	SNP	T	G
4	VIPR2		7	159043158	Silent	SNP	C	T
4	VWF		12	6052636	Missense_Mutation	SNP	C	T
4	ZNF717		3	75741293	Missense_Mutation	SNP	G	A
4	ZXDC		3	126461725	Missense_Mutation	SNP	G	C
5	ASH1L		1	155357582	Splice_Region	SNP	C	A
5	BBIP1		10	110907737	Missense_Mutation	SNP	C	T
5	BEGAIN		14	100538881	Silent	SNP	G	A
5	C2orf16		2	27580481	Silent	SNP	C	T
5	CCKBR		11	6270703	Silent	SNP	C	T
5	DISP3		1	11536339	Missense_Mutation	SNP	C	T

5	EPB41L4B		9	109256198	Silent	SNP	A	T
5	HLTF		3	149046081	Missense_Mutation	SNP	T	C
5	HTR3A		11	113989658	Silent	SNP	C	T
5	IFNA7		9	21202106	Nonsense_Mutation	SNP	G	T
5	KMT2A		11	118471722	Missense_Mutation	SNP	C	A
5	LDB1		10	102111267	Silent	SNP	C	T
5	MRPS15		1	36456213	Missense_Mutation	SNP	C	T
5	MT-ND5	MT		13215	Silent	SNP	T	C
5	PABPC1		8	100705604	Missense_Mutation	SNP	G	C
5	PLK1		16	23689250	Missense_Mutation	SNP	G	A
5	RNF20		9	101540807	Missense_Mutation	SNP	C	A
5	TIE1		1	43318049	Missense_Mutation	SNP	A	G
5	WT1		11	32396368	Frame_Shift_Ins	INS	-	T
5	WT1		11	32428521	Missense_Mutation	SNP	G	A
6	ABCC9		12	21872665	Missense_Mutation	SNP	C	T
6	ADAMTS8		11	130417004	Missense_Mutation	SNP	G	T
6	ADGRD1		12	130992343	Missense_Mutation	SNP	G	T
6	ANO8		19	17333117	Missense_Mutation	SNP	C	T
6	BCOR	X		40072832	Frame_Shift_Del	DEL	GG	-
6	BCOR	X		40072828	Missense_Mutation	SNP	G	C
6	BCOR	X		40073222	Silent	SNP	G	A
6	CES1		16	55811015	Splice_Region	SNP	A	C
6	CNTNAP1		17	42693447	Missense_Mutation	SNP	G	A
6	COG4		16	70514361	Missense_Mutation	SNP	A	C
6	DNMT3A		2	25234374	Missense_Mutation	SNP	G	A
6	FLG		1	152313941	Silent	SNP	C	T
6	IDH2		15	90088702	Missense_Mutation	SNP	C	T
6	ITGAD		16	31423955	Silent	SNP	C	T
6	ITIH5		10	7585993	Missense_Mutation	SNP	C	T
6	MAP3K21		1	233362089	Missense_Mutation	SNP	G	T
6	MUC3A		7	100958064	Silent	SNP	T	C
6	MYH14		19	50293235	Splice_Region	SNP	A	G
6	MYH6		14	23402700	Silent	SNP	G	A
6	NBPF11		1	148126846	Missense_Mutation	SNP	T	C
6	RBMXL1		1	88983751	Missense_Mutation	SNP	T	C
6	RUNX1		21	34792360	Frame_Shift_Ins	INS	-	TACA
6	SLC39A6		18	36122075	Nonsense_Mutation	SNP	G	A

6	SLC9A7	X	46759005	Missense_Mutation	SNP	G	A
6	TET2	4	105269607	Splice_Region	SNP	C	A
6	TM4SF5	17	4782947	Silent	SNP	G	T
6	TMEM230	20	5111495	Splice_Region	SNP	C	G
6	TWNK	10	100988998	Missense_Mutation	SNP	C	T
6	USH2A	1	216199844	Silent	SNP	G	A
6	ZNF443	19	12432437	Missense_Mutation	SNP	A	C
6	ZNF527	19	37389508	Nonsense_Mutation	SNP	C	T
7	ANOS1	X	8534348	Missense_Mutation	SNP	G	A
7	AP000346.1	22	23638745	Splice_Region	SNP	A	G
7	BTNL8	5	180948376	Splice_Site	DEL	GTAA	-
7	CDC14C	7	48924659	Missense_Mutation	SNP	A	C
7	CEP55	10	93515502	Missense_Mutation	SNP	C	T
7	EIF2S1	14	67364944	Silent	SNP	C	A
7	ERO1B	1	236249930	Missense_Mutation	SNP	T	A
7	FAM205A	9	34726421	Silent	SNP	T	C
7	FAM205C	9	34893049	Frame_Shift_Del	DEL	C	-
7	FAM205C	9	34893050	Silent	SNP	C	G
7	FAM47C	X	37009283	Silent	SNP	A	G
7	FAT3	11	92798031	Missense_Mutation	SNP	A	T
7	FLT3	13	28034103	In_Frame_Ins	INS	-	AAACTCCCATTTGAG<br>ATC
7	FRYL	4	48570924	Splice_Region	SNP	C	A
7	GPAT2	2	96026020	Splice_Region	SNP	A	G
7	HCN2	19	610364	Nonsense_Mutation	SNP	G	T
7	HPS4	22	26477042	Missense_Mutation	SNP	C	T
7	HSPB9	17	42122855	Missense_Mutation	SNP	A	C
7	HUNK	21	31995916	Missense_Mutation	SNP	G	A
7	IDH1	2	208248388	Missense_Mutation	SNP	C	T
7	KAT6B	10	74843084	Missense_Mutation	SNP	A	T
7	KLF17	1	44129713	Missense_Mutation	SNP	G	A
7	LACTBL1	1	22958736	Missense_Mutation	SNP	C	T
7	MT-ND5	MT	13910	Missense_Mutation	SNP	T	C
7	NECAB2	16	84002331	Silent	SNP	G	A
7	NETO1	18	72749064	Missense_Mutation	SNP	G	T
7	NPM1	5	171410539	Frame_Shift_Ins	INS	-	TCTG

7	SCN4A		17	63972629	Silent	SNP	G	A
7	SEC63		6	107921916	Splice_Region	DEL	AAAAACA	-
7	SIRPA		20	1915243	Missense_Mutation	SNP	G	C
7	SLC35F4		14	57589408	Missense_Mutation	SNP	A	G
7	TERB2		15	44978530	Missense_Mutation	SNP	C	G
7	TTN		2	178731365	Missense_Mutation	SNP	C	T
7	USP9X	X		41205346	Missense_Mutation	SNP	A	T
8	ATP5F1A		18	46084588	Missense_Mutation	SNP	A	C
8	BCOR	X		40064602	Splice_Region	SNP	G	C
8	CDH24		14	23048101	Missense_Mutation	SNP	G	A
8	FRG2C		3	75665551	Missense_Mutation	SNP	A	G
8	IFI30		19	18175205	Missense_Mutation	SNP	C	A
8	KCNK12		2	47521153	Silent	SNP	A	G
8	KRAS		12	25245347	Missense_Mutation	SNP	C	T
8	MT-CO1	MT		7278	Missense_Mutation	SNP	T	C
8	REXO1L8P		8	85644288	Splice_Region	SNP	T	C
8	SPACA1		6	88048067	Silent	SNP	C	T
8	SPI1		11	47355352	Missense_Mutation	SNP	G	C
8	TATDN1		8	124539044	Translation_Start_Site	SNP	C	A
8	TET2		4	105242908	Missense_Mutation	SNP	G	T
8	WDR17		4	176125101	Splice_Region	SNP	C	A
8	ZNF831		20	59192713	Missense_Mutation	SNP	C	A
9	AOC1		7	150857827	Silent	SNP	C	A
9	C19orf67		19	14085292	Splice_Site	SNP	C	A
9	CERS4		19	8257062	Silent	SNP	T	C
9	CNTNAP4		16	76538148	Missense_Mutation	SNP	G	A
9	DDOST		1	20660985	Missense_Mutation	SNP	C	A
9	DNAH8		6	38848683	Nonsense_Mutation	SNP	G	A
9	DNMT3A		2	25234373	Missense_Mutation	SNP	C	T
9	EVPL		17	76021492	Silent	SNP	C	G
9	GMPS		3	155936492	Silent	SNP	C	A
9	KRT76		12	52768989	In_Frame_Del	DEL	ACT	-
9	MT-ND4	MT		11823	Missense_Mutation	SNP	T	C
9	MUC3A		7	100956395	Missense_Mutation	SNP	C	G
9	MUC3A		7	100956403	Missense_Mutation	DNP	CC	TA
9	PCDHB10		5	141194472	Silent	SNP	C	G
9	PRAMEF17		1	13391965	Missense_Mutation	DNP	AA	GG

9	RHPN2P1		15	20248661	Splice_Region	SNP	A	G
9	RNPEP		1	201989500	Missense_Mutation	SNP	G	A
9	SHISAL2A		1	52656816	Missense_Mutation	SNP	C	A
9	TMC7		16	19059445	Missense_Mutation	SNP	C	A
9	TNRC6C		17	78087061	Missense_Mutation	SNP	C	A
9	TPTE2		13	19492854	Missense_Mutation	SNP	T	C
9	WDR43		2	28894728	Missense_Mutation	SNP	C	A
10	ACSL5		10	112425488	Splice_Region	SNP	T	C
10	AHI1		6	135323276	Missense_Mutation	SNP	T	C
10	BDNF		11	27658427	Silent	SNP	C	A
10	CEBPA		19	33301516	Missense_Mutation	SNP	C	A
10	CHPF2		7	151234182	Silent	SNP	C	G
10	CSMD3		8	112503979	Splice_Region	INS	-	A
10	CUEDC2		10	102423486	Silent	SNP	G	A
10	EPAS1		2	46356144	Splice_Region	TNP	TTT	CCC
10	GHR		5	42695027	Missense_Mutation	SNP	G	T
10	GRIA2		4	157221668	Splice_Region	SNP	G	T
10	GRK7		3	141816716	Missense_Mutation	SNP	A	G
10	GTF3C3		2	196797802	Missense_Mutation	SNP	T	C
10	KRBA1		7	149733826	Missense_Mutation	SNP	C	A
10	LCE1B		1	152812741	Missense_Mutation	DNP	CC	AG
10	LTF		3	46439467	Missense_Mutation	SNP	C	G
10	MBNL1		3	152414972	Missense_Mutation	SNP	T	G
10	MT-ND4	MT		11711	Missense_Mutation	SNP	G	A
10	MT-ND4	MT		11617	Silent	SNP	T	C
10	MYH2		17	10526754	Silent	SNP	G	A
10	NOTCH2NLC		1	149390829	Silent	SNP	C	A
10	NRDC		1	51800678	Silent	SNP	C	T
10	PDZRN4		12	41573038	Missense_Mutation	SNP	C	A
10	PLEC		8	143934392	Silent	SNP	A	G
10	PRRT4		7	128358781	Silent	SNP	C	G
10	PRSS3		9	33796674	Missense_Mutation	DNP	TG	CA
10	ROS1		6	117394263	Missense_Mutation	SNP	C	A
10	SAP130		2	128013100	Missense_Mutation	SNP	G	A
10	SEMA3A		7	83961514	Missense_Mutation	SNP	C	A
10	SUPT16H		14	21362929	Silent	SNP	C	A
10	SYT2		1	202596820	Silent	SNP	G	A

10	TDRD6	6	46688636	Missense_Mutation	SNP	G	A
10	TET2	4	105236901	Frame_Shift_Ins	INS	-	G
10	TMEM161B	5	88206504	Splice_Region	DEL	G	-
10	TPTE2P1	13	24961746	Splice_Region	DEL	A	-
10	WRN	8	31091892	Missense_Mutation	SNP	T	C
11	AHCTF1	1	246885532	Missense_Mutation	SNP	T	C
11	CAMK2A	5	150252066	Splice_Site	SNP	C	T
11	CHIT1	1	203222283	Silent	SNP	G	A
11	DENND1A	9	123383918	Splice_Region	SNP	C	G
11	DRC1	2	26402125	Missense_Mutation	SNP	C	T
11	EPC1	10	32293013	Missense_Mutation	SNP	C	T
11	GATA2	3	128486369	Splice_Site	SNP	C	T
11	IDH2	15	90088702	Missense_Mutation	SNP	C	T
11	KCNB2	8	72568212	Nonsense_Mutation	SNP	C	T
11	MRGPRD	11	68980465	Silent	SNP	C	A
11	MT-ND5	MT	12406	Missense_Mutation	SNP	G	A
11	MUC16	19	8967016	Silent	SNP	G	A
11	MUC3A	7	100959436	Missense_Mutation	DNP	AT	GC
11	NEB	2	151680818	Missense_Mutation	SNP	C	T
11	NEK10	3	27256296	Missense_Mutation	SNP	C	A
11	NPM1	5	171410539	Frame_Shift_Ins	INS	-	TCTG
11	NRG2	5	139852953	Missense_Mutation	DNP	TT	GA
11	OR1S2	11	58204169	Missense_Mutation	DNP	AC	CA
11	OR2T33	1	248273225	Missense_Mutation	SNP	A	C
11	RBM47	4	40432720	Silent	SNP	G	A
11	REXO1L8P	8	85644288	Splice_Region	SNP	T	C
11	SLC46A3	13	28713083	Silent	SNP	T	G
11	ST14	11	130189803	Missense_Mutation	SNP	G	A
11	TMEFF2	2	191956277	Missense_Mutation	SNP	T	G
11	TMPRSS15	21	18398225	Missense_Mutation	SNP	C	T
11	VCAN	5	83522232	Missense_Mutation	SNP	C	T
11	ZNF165	6	28085704	Missense_Mutation	SNP	G	A
11	ZNF469	16	88433467	Silent	SNP	C	T
12	ADGRL1	19	14162631	Missense_Mutation	SNP	C	A
12	ANKRD36BP2	2	88788434	Splice_Region	SNP	C	A
12	ASIC4	2	219514980	Missense_Mutation	SNP	G	A
12	CDC5L	6	44445697	Missense_Mutation	SNP	A	G

12	CLCA1	1	86493604	Splice_Region	SNP	G	A
12	COL6A3	2	237325609	Silent	SNP	G	A
12	CYP11B2	8	142911987	Missense_Mutation	SNP	A	C
12	DNAH2	17	7786910	Missense_Mutation	SNP	C	A
12	ERAP2	5	96883817	Missense_Mutation	SNP	C	A
12	FBXO15	18	74135874	Splice_Region	SNP	A	C
12	FMR1NB	X	148024873	Missense_Mutation	SNP	C	A
12	FRG2C	3	75665551	Missense_Mutation	SNP	A	G
12	GLG1	16	74532130	Silent	SNP	G	A
12	KMT5C	19	55346633	Missense_Mutation	SNP	C	A
12	KRT74	12	52573466	Missense_Mutation	SNP	C	A
12	MAP7D3	X	136230875	Missense_Mutation	SNP	T	G
12	MT-CYB	MT	15530	Silent	SNP	T	C
12	MVB12A	19	17420412	Splice_Site	SNP	G	T
12	MVD	16	88656208	Missense_Mutation	SNP	A	G
12	NSD3	8	38281568	Missense_Mutation	SNP	C	T
12	NUDT12	5	103559236	Missense_Mutation	SNP	C	T
12	PIF1	15	64817950	Missense_Mutation	SNP	C	T
12	POSTN	13	37586260	Silent	SNP	G	A
12	PRR12	19	49596741	Silent	SNP	C	G
12	SGCE	7	94628240	Missense_Mutation	SNP	G	T
12	SPHKAP	2	228021841	Silent	SNP	C	T
12	STAT6	12	57106520	Splice_Region	SNP	T	C
12	TK1	17	78185056	Missense_Mutation	SNP	G	A
12	USP6	17	5169038	Missense_Mutation	SNP	T	G
12	ZNF844	19	12076690	Nonsense_Mutation	SNP	G	T
13	ADAMTSL3	15	83983115	Missense_Mutation	SNP	C	A
13	CFAP300	11	102047896	Missense_Mutation	SNP	G	C
13	CLDN15	7	101237570	Silent	SNP	A	T
13	CLEC4C	12	7746331	Missense_Mutation	SNP	C	A
13	FMN2	1	240093522	Silent	SNP	C	A
13	FSTL5	4	161538235	Missense_Mutation	SNP	C	T
13	GRK6	5	177436152	Silent	SNP	C	T
13	HRH3	20	62216197	Missense_Mutation	SNP	C	A
13	KIF24	9	34310881	Missense_Mutation	SNP	C	A
13	LCE1A	1	152827743	Missense_Mutation	DNP	CC	AG
13	LCE1A	1	152827748	Silent	SNP	G	T

13	LEXM		1	54811790	Splice_Region	SNP	A	C
13	MPZ		1	161305973	Missense_Mutation	SNP	G	A
13	MT-CYB	MT		15200	Missense_Mutation	SNP	G	A
13	MT-ND4L	MT		10531	Missense_Mutation	SNP	T	C
13	MUC3A		7	100956403	Missense_Mutation	DNP	CC	TA
13	NKPD1		19	45153574	Missense_Mutation	SNP	C	A
13	NKTR		3	42643996	Silent	SNP	C	A
13	PALM		19	746649	Missense_Mutation	SNP	C	A
13	PLEKHA5		12	19347153	Missense_Mutation	SNP	C	A
13	POSTN		13	37583964	Splice_Region	SNP	C	T
13	RBPJ		4	26424396	Missense_Mutation	SNP	C	G
13	RXFP4		1	155941921	Missense_Mutation	SNP	C	A
13	TECTB		10	112286200	Missense_Mutation	SNP	G	A
13	TRIOBP		22	37710519	Silent	SNP	C	A
13	TRIOBP		22	37754972	Silent	SNP	C	T
13	XPO6		16	28104658	Missense_Mutation	SNP	G	T
13	ZNF687		1	151288325	Nonsense_Mutation	SNP	C	A
14	AKR1C1		10	4972686	Silent	SNP	A	G
14	ANK2		4	113356595	Silent	SNP	G	A
14	DGKD		2	233441940	Missense_Mutation	SNP	A	G
14	EXOC6B		2	72480710	Missense_Mutation	SNP	G	A
14	FAM131C		1	16058503	Missense_Mutation	DNP	GG	CA
14	GEN1		2	17761589	Splice_Region	SNP	A	G
14	IGSF3		1	116603941	Missense_Mutation	SNP	G	A
14	IRAG1		11	10626155	Silent	SNP	G	A
14	KIT		4	54733155	Missense_Mutation	SNP	A	T
14	KLHL20		1	173775681	Missense_Mutation	SNP	A	G
14	KLKB1		4	186238320	Missense_Mutation	SNP	G	A
14	NRAS		1	114716126	Missense_Mutation	SNP	C	T
14	PITPNM1		11	67499962	Missense_Mutation	SNP	C	T
14	PTCHD4		6	48009046	Silent	SNP	C	T
14	RPL3		22	39314206	Splice_Region	SNP	A	G
14	RRH		4	109844157	Missense_Mutation	SNP	T	C
14	SCARA5		8	27987610	Missense_Mutation	SNP	C	A
14	SHOX2		3	158105051	Splice_Site	DEL	AACACA	-
14	SON		21	33559643	Missense_Mutation	SNP	C	A
14	SPRR2E		1	153093591	Missense_Mutation	SNP	C	T

14	SPRR2E	1	153093596	Silent	SNP	T	C
15	ABAT	16	8775024	Silent	SNP	C	A
15	ABCB4	7	87450090	Splice_Region	SNP	T	A
15	AIG1	6	143334100	Splice_Region	SNP	G	A
15	ANKRD36B	2	97560842	Missense_Mutation	SNP	C	G
15	CELA3B	1	21980847	Silent	SNP	C	T
15	CERS6	2	168561259	Missense_Mutation	SNP	G	T
15	CLCNKA	1	16026766	Splice_Site	DEL	CCCTTCAGCGGTGA GACCCCCCTCATGC	-
15	CSPG4	15	75689448	Silent	SNP	T	A
15	FMO3	1	171116275	Silent	SNP	C	A
15	FRG2C	3	75664415	Silent	SNP	T	C
15	GGT7	20	34854622	Splice_Region	SNP	G	C
15	HCAR3	12	122715787	Missense_Mutation	SNP	T	C
15	IDH1	2	208248388	Missense_Mutation	SNP	C	T
15	IL1RN	2	113120136	Splice_Region	SNP	A	C
15	IVL	1	152911132	Silent	SNP	G	A
15	KLHL30	2	238140899	Missense_Mutation	SNP	G	A
15	MAMDC4	9	136854615	Silent	SNP	C	T
15	MINAR1	15	79463260	Missense_Mutation	SNP	C	A
15	MMAB	12	109557065	Missense_Mutation	SNP	A	T
15	MT-CYB	MT	14770	Silent	SNP	C	T
15	MT-ND4	MT	11253	Missense_Mutation	SNP	T	C
15	MT-ND4	MT	11372	Missense_Mutation	SNP	G	A
15	MUC3A	7	100959497	Missense_Mutation	SNP	T	C
15	NPM1	5	171410539	Frame_Shift_Ins	INS	-	TCTG
15	OR1L6	9	122750491	Missense_Mutation	SNP	T	C
15	OR5H1	3	98133124	Missense_Mutation	SNP	C	T
15	OR8B3	11	124396494	Silent	SNP	A	G
15	PCDHB9	5	141189223	Silent	SNP	G	A
15	PCDHGA1	5	141332050	Missense_Mutation	SNP	T	C
15	PDE4DIP	1	149029906	Silent	SNP	G	A
15	PLIN4	19	4512214	Silent	SNP	A	G
15	PSMA6	14	35317262	Missense_Mutation	SNP	G	A
15	REM2	14	22886902	Missense_Mutation	SNP	T	A
15	RERE	1	8358590	Silent	SNP	T	C
15	SLC34A2	4	25662568	Missense_Mutation	SNP	G	A

15	TBC1D5		3	17166775	Missense_Mutation	SNP	T	C
15	TNN		1	175128626	Nonsense_Mutation	SNP	C	A
15	ZNF705G		8	7361230	Missense_Mutation	SNP	G	C
16	AEBP1		7	44113999	Missense_Mutation	SNP	C	A
16	AHNAK2		14	104947873	Silent	SNP	G	C
16	ARRDC5		19	4891344	Missense_Mutation	SNP	C	G
16	ASMTL	X		1418024	Missense_Mutation	SNP	C	T
16	BCOR	X		40070979	Frame_Shift_Del	DEL	C	-
16	CACNA1I		22	39662796	Silent	SNP	C	T
16	CBL		11	119278170	Missense_Mutation	SNP	A	G
16	CHAF1B		21	36402796	Silent	SNP	G	A
16	CLTC		17	59693730	Missense_Mutation	SNP	C	G
16	CORO6		17	29617509	Silent	SNP	C	A
16	DNMT3A		2	25234343	Nonsense_Mutation	SNP	G	C
16	DPF3		14	72671166	Silent	SNP	C	T
16	ESPNL		2	238131240	Silent	SNP	C	T
16	FAM200B		4	15687685	Silent	SNP	A	C
16	FAM90A28P		19	53300877	Splice_Region	SNP	G	T
16	FGFRL1		4	1024917	Missense_Mutation	SNP	C	A
16	FLT3		13	28035621	Missense_Mutation	SNP	C	G
16	IGFL1		19	46230449	Silent	SNP	C	T
16	KMT2B		19	35730845	Silent	SNP	G	C
16	KRT32		17	41463025	Missense_Mutation	SNP	G	A
16	LAMA2		6	129452984	Splice_Region	SNP	A	T
16	LRP1		12	57180137	Silent	SNP	C	A
16	LRRC7		1	70038681	Missense_Mutation	SNP	C	T
16	LYZL2		10	30629668	Missense_Mutation	SNP	T	C
16	MAP1A		15	43524787	Missense_Mutation	SNP	C	A
16	MEGF6		1	3499585	Splice_Region	SNP	C	T
16	MOCS3		20	50959561	Missense_Mutation	SNP	C	T
16	MT-CO1	MT		6587	Silent	SNP	C	G
16	MT-CO3	MT		9305	Missense_Mutation	SNP	G	C
16	MTHFSD		16	86532276	Missense_Mutation	SNP	G	C
16	MT-ND2	MT		5353	Missense_Mutation	SNP	G	C
16	MT-ND4	MT		11888	Missense_Mutation	SNP	G	C
16	MUC5B		11	1237056	Silent	SNP	C	T
16	MYH9		22	36295575	Missense_Mutation	SNP	C	T

16	NRXN1		2	50465537	Missense_Mutation	SNP	T	C
16	NRXN2		11	64653719	Missense_Mutation	SNP	C	A
16	PARP4		13	24447125	Missense_Mutation	SNP	T	C
16	PCNX3		11	65619907	Silent	SNP	T	A
16	PDE6A		5	149895281	Missense_Mutation	SNP	G	A
16	PEG3		19	56813711	Silent	SNP	C	A
16	PLIN4		19	4512617	Missense_Mutation	SNP	C	T
16	PRDM9		5	23524350	Missense_Mutation	SNP	C	T
16	RALGAPA1		14	35683971	Missense_Mutation	SNP	T	C
16	RBMX	X		136875371	Silent	SNP	T	C
16	RUNX1		21	34880640	In_Frame_Ins	INS	-	CCGGCCCAC
16	SEMA6A		5	116447056	Missense_Mutation	SNP	G	A
16	SLC26A9		1	205935797	Silent	SNP	G	A
16	STAG2	X		124051377	Frame_Shift_Del	DEL	ACTC	-
16	STARD13		13	33127545	Nonsense_Mutation	SNP	G	A
16	SYN2		3	12169844	Missense_Mutation	SNP	C	A
16	TBC1D5		3	17372214	Missense_Mutation	SNP	C	T
16	TYW1B		7	72575573	Frame_Shift_Del	DEL	G	-
16	WAC		10	28589823	Missense_Mutation	SNP	T	A
16	ZNF839		14	102331818	Missense_Mutation	SNP	C	A
17	ANKRD36		2	97162115	Missense_Mutation	SNP	C	T
17	BRPF1		3	9743843	Missense_Mutation	SNP	C	A
17	CNOT2		12	70337401	Missense_Mutation	SNP	C	A
17	DISP2		15	40363644	Missense_Mutation	SNP	C	G
17	DNAAF5		7	770500	Missense_Mutation	SNP	G	A
17	DNAH5		5	13859522	Missense_Mutation	SNP	G	A
17	DTX4		11	59182302	Missense_Mutation	SNP	C	T
17	FLG2		1	152354993	Silent	SNP	C	T
17	H2AC13		6	27808224	Silent	SNP	C	T
17	KRTAP10-2		21	44551006	Missense_Mutation	DNP	GC	AT
17	MSL2		3	136151639	Missense_Mutation	SNP	A	C
17	MT-CO3	MT		9570	Missense_Mutation	SNP	A	C
17	MUC5AC		11	1190539	Missense_Mutation	DNP	GC	TT
17	MUC5AC		11	1190543	Missense_Mutation	SNP	C	A
17	NISCH		3	52478097	Missense_Mutation	SNP	C	A
17	OR10G7		11	124038920	Missense_Mutation	SNP	T	C

17	OR8G5	11	124265801	Silent	SNP	C	T
17	PLCH1	3	155596245	Silent	SNP	A	G
17	POLR1A	2	86054150	Missense_Mutation	SNP	C	A
17	SELENOS	15	101277263	Missense_Mutation	SNP	C	A
17	SLC19A1	21	45515778	Frame_Shift_Ins	INS	-	GCAGA
17	TACC1	8	38819659	Missense_Mutation	SNP	C	G
17	TPRN	9	137199029	Silent	SNP	C	A
17	TRAPPC9	8	140371035	Missense_Mutation	SNP	G	A
17	TXNDC5	6	7888855	Splice_Region	SNP	A	G
18	ATP10A	15	25680275	Missense_Mutation	SNP	C	A
18	CAMK2A	5	150251743	Splice_Region	SNP	C	A
18	CAMK2A	5	150251749	Splice_Site	SNP	C	A
18	CCR10	17	42680192	Silent	SNP	C	A
18	CDK5RAP1	20	33367014	Splice_Region	DEL	A	-
18	CYP2A6	19	40848629	Missense_Mutation	SNP	G	T
18	CYP2A6	19	40848633	Missense_Mutation	SNP	G	C
18	ELL2	5	95900688	Splice_Region	SNP	C	A
18	EPYC	12	90972827	Missense_Mutation	SNP	C	A
18	EXOC6B	2	72741337	Silent	SNP	C	A
18	GBF1	10	102376672	Missense_Mutation	SNP	C	G
18	GIMAP1	7	150720704	Missense_Mutation	SNP	G	C
18	HMGXB4	22	35264694	Missense_Mutation	SNP	C	A
18	LIPT1	2	99162760	Missense_Mutation	SNP	C	G
18	LZTR1	22	20982389	Missense_Mutation	SNP	C	A
18	MFSD13A	10	102471053	Missense_Mutation	SNP	C	A
18	MID1	X	10567335	Missense_Mutation	SNP	G	C
18	MUC5AC	11	1190028	Silent	SNP	G	A
18	MUC5B	11	1247701	Silent	SNP	A	C
18	NME4	16	399487	Splice_Region	SNP	C	A
18	NUP205	7	135619452	Missense_Mutation	SNP	A	C
18	OR2T8	1	247921552	Missense_Mutation	SNP	A	G
18	PCNA	20	5118604	Splice_Region	DEL	ACTC	-
18	PKHD1L1	8	109465237	Nonsense_Mutation	SNP	C	G
18	PTPN11	12	112450395	Missense_Mutation	SNP	C	T
18	SHISA9	16	12902599	Missense_Mutation	SNP	C	A
18	SHPRH	6	145894243	Splice_Region	SNP	C	A
18	SPRR2E	1	153093591	Missense_Mutation	SNP	C	T

18	SPRR2E		1	153093596	Silent	SNP	T	C
18	SPTAN1		9	128587675	Missense_Mutation	SNP	C	G
18	TMEM30B		14	61280255	Missense_Mutation	SNP	C	A
18	TSPYL2	X		53084860	Missense_Mutation	SNP	C	A
18	TTC30A		2	177617882	Missense_Mutation	SNP	G	A
18	TYW1		7	67083465	Nonsense_Mutation	SNP	G	A
18	TYW1		7	67083457	Silent	SNP	G	A
18	UBE2W		8	73878893	Missense_Mutation	SNP	C	A
18	UBFD1		16	23558082	Missense_Mutation	SNP	G	A
18	UBFD1		16	23558090	Missense_Mutation	SNP	C	T
18	WLS		1	68137938	Splice_Region	SNP	G	T
18	ZNF862		7	149864280	Missense_Mutation	SNP	C	A
19	ACSL4	X		109733134	Splice_Region	SNP	C	A
19	ADGRG3		16	57685673	Nonsense_Mutation	SNP	C	A
19	CACNA1A		19	13208878	In_Frame_Del	DEL	GAT	-
19	DNAH3		16	21034071	Missense_Mutation	SNP	C	A
19	GP2		16	20317363	Silent	SNP	T	C
19	HERC2		15	28214778	Missense_Mutation	SNP	C	A
19	INTS11		1	1321045	Missense_Mutation	SNP	C	A
19	KRT17P2		17	18430783	Splice_Region	SNP	G	A
19	MUC5AC		11	1185018	Missense_Mutation	SNP	A	C
19	MUC5AC		11	1185024	Silent	SNP	C	T
19	OR2T34		1	248573992	Missense_Mutation	DNP	GC	AG
19	PFAS		17	8264906	Silent	SNP	T	C
19	POM121		7	72942042	Silent	SNP	G	A
19	SLF2		10	100929422	Silent	SNP	C	A
19	TBCEL		11	121087011	Missense_Mutation	SNP	A	T
19	THBS1		15	39588677	Nonsense_Mutation	SNP	C	A
19	TMED8		14	77377020	Missense_Mutation	SNP	A	G
19	WASHC2A		10	50093380	Silent	SNP	C	T
19	ZNF579		19	55578710	Silent	SNP	G	T
20	AGAP10P		10	45679043	Splice_Region	SNP	A	G
20	AHNAK2		14	104949024	Missense_Mutation	DNP	TG	CA
20	COL11A1		1	102962242	Missense_Mutation	SNP	G	C
20	DES		2	219420155	Splice_Region	SNP	G	A
20	DSPP		4	87616153	In_Frame_Ins	INS	-	CAGCAG
20	EP400		12	132062523	In_Frame_Ins	INS	-	GCAGCA

20	ESPN		1	6440652	Silent	SNP	C	G
20	FAM131C		1	16062107	Missense_Mutation	SNP	G	A
20	FAM160A2		11	6223988	Silent	SNP	T	C
20	FBXO38		5	148423994	Splice_Region	SNP	G	T
20	FNDC1		6	159234047	In_Frame_Del	DEL	GAC	-
20	GOLGA6L6		15	20534891	Missense_Mutation	SNP	G	A
20	GOLGA8S		15	23361379	In_Frame_Del	DEL	GAGCAGCAGGAGAG	-
20	GOLGA8S		15	23364574	Silent	SNP	G	A
20	GXYLT2		3	72922334	Missense_Mutation	SNP	C	T
20	HMGA1		6	34242734	Missense_Mutation	SNP	C	A
20	HRNR		1	152221375	Missense_Mutation	SNP	C	T
20	LRRC25		19	18396648	Missense_Mutation	SNP	C	T
20	MAML3		4	139889910	In_Frame_Del	DEL	TGC	-
20	MC4R		18	60372084	Missense_Mutation	SNP	G	C
20	MN1		22	27798031	Missense_Mutation	SNP	C	A
20	MT-CO1	MT		7211	Silent	SNP	G	A
20	MT-CO2	MT		7822	Silent	SNP	A	G
20	MT-ND3	MT		10101	Silent	SNP	T	C
20	MUC5AC		11	1189952	Missense_Mutation	SNP	G	A
20	NF1P6		22	15628374	Splice_Region	DEL	A	-
20	NKX2-3		10	99535469	Silent	SNP	C	A
20	NRAS		1	114716123	Missense_Mutation	SNP	C	T
20	NUP85		17	75205783	Missense_Mutation	SNP	C	T
20	ODF1		8	102560796	Frame_Shift_Del	DEL	GCCCCTGC	-
20	ODF1		8	102560805	Frame_Shift_Del	DEL	ACCCGTGCAG	-
20	OR8G3P		11	124214920	Missense_Mutation	SNP	C	T
20	PAK2		3	196803046	Silent	SNP	A	G
20	PLCL1		2	198085885	Nonsense_Mutation	SNP	G	T
20	PRKDC		8	47819514	Splice_Region	DEL	AA	-
20	SLC15A4		12	128808801	Silent	SNP	C	T
20	SLC6A19		5	1212320	Missense_Mutation	SNP	G	A
20	SUSD2		22	24183081	Missense_Mutation	SNP	G	A
20	TENT4B		16	50217660	Silent	SNP	C	T
20	THAP12		11	76361032	Missense_Mutation	SNP	G	A
20	THAP12		11	76361041	Missense_Mutation	SNP	C	T
20	TMEM178A		2	39717217	Missense_Mutation	SNP	C	A
20	TRPM3		9	70625523	Splice_Region	SNP	A	G

20	TTC27		2	32733873	Missense_Mutation	SNP	C	T
20	VCX	X		7843976	Missense_Mutation	SNP	C	T
20	VPS13D		1	12346645	Missense_Mutation	SNP	A	T
20	WT1		11	32396363	Frame_Shift_Ins	INS	-	GACCG
20	ZFHX4		8	76704695	Missense_Mutation	SNP	A	G
20	ZNF736		7	64349060	Nonsense_Mutation	SNP	T	A
20	ZNF860		3	31990470	Missense_Mutation	SNP	A	G
21	ACACB		12	109206855	Missense_Mutation	SNP	A	T
21	AEBP1		7	44111057	Missense_Mutation	SNP	C	T
21	AKAP3		12	4627876	Silent	SNP	G	A
21	ANK2		4	113242181	Missense_Mutation	SNP	G	C
21	ANP32A		15	68784410	Missense_Mutation	SNP	C	A
21	ARHGEF4		2	130916712	Silent	SNP	A	G
21	B4GALNT2		17	49164127	Missense_Mutation	SNP	C	A
21	BAHD1		15	40458498	Missense_Mutation	SNP	C	A
21	CACNA1A		19	13359696	Silent	SNP	G	A
21	CDRT1		17	15613970	Missense_Mutation	SNP	G	C
21	CHD8		14	21393235	Missense_Mutation	SNP	C	A
21	CMPK1		1	47333978	Missense_Mutation	SNP	C	A
21	COL4A1		13	110212610	Silent	SNP	C	T
21	CPQ		8	96965998	Missense_Mutation	SNP	G	A
21	CRACD		4	56315350	Silent	SNP	C	T
21	CRELD1		3	9943972	Silent	SNP	C	T
21	DNAH11		7	21765425	Splice_Region	SNP	C	A
21	DRC3		17	17997641	Splice_Region	SNP	C	A
21	DYSF		2	71574300	Missense_Mutation	SNP	C	T
21	EPHA2		1	16132227	Missense_Mutation	SNP	C	A
21	EPHA5		4	65490650	Missense_Mutation	SNP	A	C
21	EXO5		1	40514947	Missense_Mutation	SNP	C	A
21	FBXW4		10	101694572	Silent	SNP	G	C
21	FGFR4		5	177092714	Silent	SNP	C	T
21	FLNC		7	128840130	Missense_Mutation	SNP	G	A
21	GLI2		2	120989229	Silent	SNP	C	T
21	GRID2IP		7	6551039	Missense_Mutation	SNP	C	T
21	GYPA		4	144111395	Silent	SNP	C	T
21	HCAR2		12	122702353	Missense_Mutation	SNP	G	A
21	IGFN1		1	201210252	Missense_Mutation	SNP	T	G

21	IGLV2-18		22	22734637	Missense_Mutation	DNP	CC	AT
21	JMJD7-PLA2(		15	41841819	Missense_Mutation	SNP	C	A
21	JMJD7-PLA2(		15	41841836	Missense_Mutation	SNP	C	A
21	KCNV1		8	109968248	Missense_Mutation	SNP	C	T
21	KLF14		7	130733107	Silent	SNP	G	A
21	KRAS		12	25245347	Missense_Mutation	SNP	C	T
21	LCORL		4	17909120	Missense_Mutation	SNP	C	T
21	LGALS9C		17	18492817	Silent	SNP	T	C
21	MAP6		11	75667629	Missense_Mutation	SNP	A	C
21	MT-CYB	MT		15234	Nonsense_Mutation	SNP	G	A
21	MUC17		7	101042732	Missense_Mutation	SNP	C	A
21	NF1		17	31252934	Splice_Region	SNP	G	A
21	NFIB		9	14150139	Splice_Region	SNP	C	A
21	NIT2		3	100355212	Missense_Mutation	SNP	C	T
21	NKX6-3		8	41650334	Silent	SNP	G	A
21	NPIPB9		16	28757993	Splice_Region	DEL	A	-
21	NPIPB9		16	28757997	Splice_Site	SNP	G	T
21	OR5D13		11	55773970	Missense_Mutation	SNP	A	C
21	PCDH15		10	54329671	Silent	SNP	C	T
21	PCDHA2		5	140795264	Silent	SNP	G	A
21	PDE7A		8	65779795	Missense_Mutation	SNP	G	A
21	PDZRN3		3	73384828	Missense_Mutation	SNP	T	C
21	PXK		3	58382687	Silent	SNP	C	A
21	RAB5B		12	55990698	Missense_Mutation	SNP	C	A
21	RBM33		7	155745535	Missense_Mutation	SNP	C	A
21	RGS11		16	271092	Missense_Mutation	SNP	C	A
21	RGS11		16	271084	Silent	SNP	C	A
21	RNF26		11	119335159	Missense_Mutation	SNP	C	A
21	ROPN1B		3	125983277	Missense_Mutation	SNP	C	T
21	TRAF4		17	28749335	Missense_Mutation	SNP	C	A
21	TRPC6		11	101491657	Missense_Mutation	SNP	C	T
21	WASF3		13	26682788	Missense_Mutation	SNP	G	A
21	ZNF567		19	36720476	Silent	SNP	G	A
22	ACAN		15	88856812	Missense_Mutation	SNP	G	C
22	ACSM5		16	20439925	Splice_Region	SNP	T	C
22	AL355102.2		14	96262712	Silent	SNP	C	T
22	BAIAP3		16	1341329	Missense_Mutation	SNP	C	A

22	BAIAP3		16	1341354	Missense_Mutation	SNP	C	G
22	BCAP31	X		153703963	Missense_Mutation	SNP	T	G
22	BCAP31	X		153703954	Splice_Region	SNP	C	A
22	CCDC158		4	76353243	Missense_Mutation	SNP	A	T
22	CCDC159		19	11354640	Missense_Mutation	SNP	G	C
22	CLEC4GP1		19	7788796	Splice_Region	SNP	G	T
22	CNTNAP3		9	39287979	Splice_Site	SNP	C	T
22	COL4A1		13	110176629	Missense_Mutation	SNP	G	A
22	DAAM2		6	39856358	Missense_Mutation	SNP	G	A
22	DAZAP1		19	1418712	Missense_Mutation	SNP	C	A
22	DAZAP1		19	1418695	Silent	SNP	G	A
22	DRC1		2	26449998	Splice_Region	SNP	G	T
22	EPG5		18	45852481	Missense_Mutation	SNP	C	A
22	EPG5		18	45852495	Missense_Mutation	SNP	T	A
22	ESAM		11	124753762	Missense_Mutation	SNP	C	A
22	FAM186B		12	49600402	Missense_Mutation	SNP	A	T
22	FAM83A		8	123194037	Missense_Mutation	SNP	G	A
22	FLT3		13	28018505	Missense_Mutation	SNP	C	A
22	FRG2C		3	75664415	Silent	SNP	T	C
22	GFOD2		16	67675678	Missense_Mutation	SNP	C	A
22	GRIA1		5	153802465	Missense_Mutation	SNP	C	A
22	IDS	X		149501018	Silent	SNP	G	A
22	KRT15		17	41518389	Missense_Mutation	SNP	T	C
22	KRTAP11-1		21	30881075	Silent	SNP	C	A
22	KRTAP11-1		21	30881087	Silent	SNP	C	A
22	LARGE2		11	45927982	Missense_Mutation	DNP	TC	AT
22	LARGE2		11	45927974	Silent	SNP	G	A
22	LRRC74B		22	21049086	Missense_Mutation	DNP	CC	AT
22	LYZL2		10	30629620	Missense_Mutation	SNP	G	C
22	MAVS		20	3864513	Missense_Mutation	SNP	C	A
22	MAVS		20	3865719	Missense_Mutation	SNP	G	A
22	MAVS		20	3865726	Missense_Mutation	SNP	G	A
22	MT-ND4	MT		11351	Missense_Mutation	SNP	G	A
22	NAALADL1		11	65054269	Nonsense_Mutation	SNP	C	A
22	NBPF12		1	146994484	Missense_Mutation	SNP	C	G
22	NBPF12		1	146984882	Silent	SNP	A	G
22	NUP50		22	45178264	Missense_Mutation	SNP	A	G

22	OR11H2		14	19713450	Missense_Mutation	SNP	T	C
22	PASD1	X		151671039	Missense_Mutation	SNP	C	A
22	PASD1	X		151671044	Missense_Mutation	SNP	C	A
22	PCDHB16		5	141184356	Silent	SNP	T	C
22	PITPNM1		11	67492945	Missense_Mutation	SNP	C	A
22	PLBD1		12	14536699	Silent	SNP	C	G
22	PLIN4		19	4511936	Missense_Mutation	SNP	A	G
22	PLIN4		19	4511586	Silent	DNP	AC	GG
22	PLIN4		19	4511923	Silent	SNP	T	C
22	PRSS3		9	33796801	Missense_Mutation	SNP	A	T
22	RBM27		5	146254937	Splice_Region	SNP	C	T
22	REST		4	56931052	In_Frame_Del	DEL	GAGCTGTCTCCTCCC<br>ATGGAGGTGGTCCA<br>GAAGGAGCCTGTTC	-
22	RPTOR		17	80823209	Nonsense_Mutation	SNP	C	A
22	RSPH6A		19	45810816	Silent	SNP	C	A
22	SHANK1		19	50697837	Splice_Region	SNP	C	A
22	SLC16A8		22	38081173	Missense_Mutation	SNP	C	A
22	SPRED2		2	65344803	In_Frame_Ins	INS	-	CGGGCC
22	SSU72P2		11	4242330	Missense_Mutation	SNP	A	G
22	TRPM5		11	2414170	Missense_Mutation	SNP	C	T
22	TRPM6		9	74788618	Missense_Mutation	SNP	C	A
22	TXNRD3		3	126654829	Missense_Mutation	SNP	C	A
22	TXNRD3		3	126654835	Silent	SNP	G	T
22	UBAP1L		15	65099703	Silent	SNP	C	A
22	UGT2B28		4	69280512	Silent	SNP	G	A
22	UIMC1		5	176969229	Missense_Mutation	SNP	C	A
22	ZNF182	X		47977356	Missense_Mutation	SNP	C	A
22	ZNF189		9	101408446	Missense_Mutation	SNP	C	A
22	ZNF761		19	53454872	Missense_Mutation	SNP	T	G
23	AC099489.1		16	11446863	Missense_Mutation	SNP	G	T
23	ACSM4		12	7326979	Missense_Mutation	SNP	G	A
23	ARMC3		10	23008847	Missense_Mutation	SNP	G	C
23	ATP1A1		1	116395130	Missense_Mutation	SNP	C	A
23	BAHCC1		17	81444699	Frame_Shift_Ins	INS	-	C
23	CCAR1		10	68722574	Missense_Mutation	SNP	C	A
23	COMTD1		10	75235619	Missense_Mutation	SNP	C	A

23	CSMD1	8	3029478	Nonsense_Mutation	SNP	C	A
23	CTCF	16	67611172	Frame_Shift_Del	DEL	C	-
23	DACT2	6	168308136	Missense_Mutation	SNP	T	G
23	DDX17	22	38487915	Frame_Shift_Ins	INS	-	C
23	DNAH17	17	78426576	Missense_Mutation	SNP	C	A
23	DNAH9	17	11871737	Missense_Mutation	SNP	G	A
23	DNHD1	11	6571706	Missense_Mutation	SNP	C	A
23	DNMT3A	2	25235779	Missense_Mutation	SNP	T	C
23	ELFN2	22	37373596	Missense_Mutation	SNP	C	A
23	EPG5	18	45870696	Missense_Mutation	SNP	C	A
23	FAM153A	5	177737043	Missense_Mutation	SNP	G	A
23	FBL	19	39840639	Silent	SNP	G	A
23	FRG1JP	9	63837727	Splice_Region	SNP	T	A
23	LGALS9C	17	18492817	Silent	SNP	T	C
23	LRRN3	7	111124423	Missense_Mutation	SNP	A	T
23	MBD6	12	57527190	Missense_Mutation	SNP	C	A
23	MIOX	22	50488339	Silent	SNP	C	T
23	MT-CO1	MT	6580	Missense_Mutation	SNP	G	A
23	MYO18B	22	26004827	Missense_Mutation	SNP	G	A
23	MYOM3	1	24092278	Silent	SNP	T	C
23	NBPF26	1	120824037	Silent	SNP	T	C
23	NMD3	3	161235142	Missense_Mutation	SNP	C	A
23	NPM1	5	171410539	Frame_Shift_Ins	INS	-	TCTG
23	OBSCN	1	228377223	Missense_Mutation	SNP	C	A
23	OPTN	10	13124003	Silent	SNP	C	T
23	OR10G4	11	124015613	Silent	SNP	A	G
23	OR2T8	1	247921607	Missense_Mutation	SNP	T	G
23	OR2T8	1	247921678	Missense_Mutation	SNP	G	T
23	PAPPA2	1	176740130	Missense_Mutation	SNP	G	A
23	PRAMEF2	1	12861684	Missense_Mutation	SNP	T	G
23	PTPN11	12	112453279	Missense_Mutation	SNP	G	C
23	RBMX	X	136876656	Splice_Site	INS	-	CATG
23	RYR1	19	38451842	Missense_Mutation	SNP	C	T
23	SERPINA9	14	94469429	Missense_Mutation	SNP	C	T
23	SH2D7	15	78098579	Missense_Mutation	SNP	C	G
23	SLC22A10	11	63291641	Missense_Mutation	SNP	C	A
23	SORBS2	4	185624125	Missense_Mutation	SNP	C	A

23	SRD5A2	2	31533759	Missense_Mutation	SNP	C	A
23	TET2	4	105243621	Missense_Mutation	SNP	C	G
23	TET2	4	105235572	Nonsense_Mutation	SNP	C	T
23	TTN	2	178776988	Missense_Mutation	SNP	C	G
23	USH2A	1	215680212	Silent	SNP	T	C
23	USP6NL	10	11463744	Missense_Mutation	SNP	G	A
23	YPEL4	11	57646360	Silent	SNP	C	A
23	ZNF417	19	57909712	Missense_Mutation	SNP	G	T
23	ZNF462	9	106928971	Nonsense_Mutation	SNP	C	T
24	ABCA13	7	48520070	Silent	SNP	G	A
24	ABCA13	7	48520100	Silent	SNP	T	C
24	ABCA13	7	48524317	Silent	SNP	A	G
24	ABCB1	7	87509329	Silent	SNP	A	G
24	ABCB5	7	20658647	Missense_Mutation	SNP	A	G
24	ABCB5	7	20643581	Silent	SNP	G	T
24	AC010255.2	5	122182614	Missense_Mutation	SNP	G	A
24	ACCSL	11	44058660	Missense_Mutation	SNP	T	C
24	ADAM22	7	88145412	Splice_Region	SNP	T	C
24	ADRB2	5	148827083	Silent	SNP	G	A
24	ADRB2	5	148827354	Silent	SNP	C	A
24	AGR2	7	16794973	Silent	SNP	A	G
24	AK2	1	33036775	Silent	SNP	G	A
24	AKAP9	7	92022864	In_Frame_Ins	INS	-	AAC
24	AKAP9	7	92062287	Silent	SNP	C	T
24	AKAP9	7	92077883	Splice_Region	SNP	C	T
24	ALPK2	18	58536179	Silent	SNP	G	C
24	ANKMY2	7	16627116	Silent	SNP	T	G
24	ANKMY2	7	16627134	Silent	SNP	G	C
24	ANKRD31	5	75195891	In_Frame_Del	DEL	TCA	-
24	ANKRD36C	2	95951354	Missense_Mutation	SNP	C	T
24	ANLN	7	36419467	Silent	SNP	G	A
24	AP000311.1	21	33912376	Missense_Mutation	SNP	A	C
24	AP000311.1	21	33912381	Missense_Mutation	SNP	C	T
24	AP4M1	7	100106825	Silent	SNP	C	T
24	APC	5	112840862	Silent	SNP	T	G
24	APOBEC3F	22	39045091	Missense_Mutation	SNP	G	T
24	ARHGEF28	5	73849020	Silent	SNP	A	G

24	ASB3	2	53700281	Nonsense_Mutation	INS	-	AAACCAGCGAACTTG GCTAGCTTGCCAAAC G
24	ASGR1	17	7174446	In_Frame_Ins	INS	-	TTCGTGTCTGAC
24	ATP8B1	18	57655270	Missense_Mutation	SNP	C	T
24	ATP9B	18	79307183	Silent	SNP	C	T
24	BAIAP2L1	7	98293539	Silent	SNP	C	T
24	BAIAP2L1	7	98293563	Silent	SNP	G	A
24	BAZ2A	12	56613214	Silent	SNP	T	C
24	BIRC6	2	32491544	Missense_Mutation	SNP	C	A
24	BMPER	7	33970334	Silent	SNP	C	T
24	BPI	20	38318446	Missense_Mutation	SNP	G	A
24	BTBD16	10	122336546	Missense_Mutation	SNP	A	C
24	C1D	2	68042936	Missense_Mutation	SNP	A	G
24	C7orf31	7	25155046	Missense_Mutation	SNP	G	C
24	C7orf57	7	48049974	Missense_Mutation	SNP	C	A
24	C7orf57	7	48049945	Silent	SNP	T	C
24	CADPS2	7	122438360	Silent	SNP	T	G
24	CARD6	5	40841639	Missense_Mutation	SNP	C	T
24	CCDC178	18	33266931	Missense_Mutation	SNP	A	T
24	CDC20B	5	55124971	Silent	SNP	A	C
24	CDH20	18	61550172	Missense_Mutation	SNP	G	C
24	CDH26	20	60001394	Missense_Mutation	SNP	T	G
24	CDK5RAP1	20	33394043	Silent	SNP	T	C
24	CEACAM1	19	42527255	Silent	SNP	C	G
24	CFAP69	7	90309366	Missense_Mutation	SNP	C	T
24	CFTR	7	117559479	Missense_Mutation	SNP	G	A
24	CLCN1	7	143345744	Silent	SNP	C	T
24	CNTNAP2	7	147977886	Silent	SNP	A	G
24	COMP	19	18785055	Silent	SNP	C	T
24	COPG2	7	130508666	Splice_Region	SNP	G	A
24	CTTNBP2	7	117735320	Missense_Mutation	SNP	A	C
24	CYFIP2	5	157326249	Silent	SNP	C	T
24	DCHS2	4	154236428	Missense_Mutation	SNP	C	A
24	DDC	7	50476616	Splice_Region	SNP	C	T

24	DDX11	12	31089424	Missense_Mutation	SNP	G	A
24	DDX11	12	31089428	Missense_Mutation	SNP	A	G
24	DDX27	20	49233645	Silent	SNP	C	T
24	DHX33	17	5463627	Missense_Mutation	SNP	G	A
24	DKKL1	19	49364656	Missense_Mutation	SNP	G	A
24	DNAH11	7	21558946	Missense_Mutation	SNP	A	G
24	DNAH11	7	21738831	Silent	SNP	C	T
24	ERAP1	5	96793832	Missense_Mutation	SNP	T	C
24	EXOC3L4	14	103102392	Silent	SNP	A	G
24	EXOC4	7	133895694	Silent	SNP	G	A
24	FAM170A	5	119634265	Missense_Mutation	SNP	C	T
24	FAM71B	5	157162574	Missense_Mutation	SNP	A	G
24	FCGBP	19	39877690	Missense_Mutation	SNP	C	T
24	FECH	18	57554416	Silent	SNP	T	C
24	FEZF1	7	122302793	Splice_Region	SNP	G	C
24	FHOD3	18	36652647	Missense_Mutation	SNP	C	T
24	FLG	1	152306079	Missense_Mutation	SNP	T	C
24	FLG	1	152307556	Missense_Mutation	SNP	T	C
24	FMC1-LUC7L	7	139341406	Missense_Mutation	SNP	T	G
24	FOXP4	6	41565836	Missense_Mutation	SNP	C	A
24	FREM1	9	14746362	Missense_Mutation	SNP	C	A
24	FRY	13	32228535	Missense_Mutation	SNP	C	A
24	FYB1	5	39119621	Missense_Mutation	SNP	C	A
24	GCC1	7	127582103	Silent	SNP	G	A
24	GCC1	7	127584232	Silent	SNP	A	G
24	GCNT4	5	75028723	Missense_Mutation	SNP	G	A
24	HAPLN3	15	88878096	Silent	SNP	A	G
24	HBP1	7	107185936	Silent	SNP	C	T
24	HDAC9	7	18727720	Silent	SNP	A	G
24	HIPK3	11	33351653	Silent	SNP	C	A
24	HUS1	7	47965365	Silent	SNP	C	T
24	IGFN1	1	201209837	Missense_Mutation	SNP	A	G
24	IGKV1-16	2	89100147	Silent	SNP	T	C
24	IGLV5-48	22	22352950	Missense_Mutation	SNP	C	A
24	IMMT	2	86144547	Silent	SNP	C	A
24	IMPG1	6	75931086	Missense_Mutation	SNP	G	A
24	IRAK1BP1	6	78885380	Splice_Region	SNP	A	G

24	ITGA1	5	52897456	Splice_Region	SNP	C	T
24	ITGB8	7	20379303	Splice_Region	SNP	T	C
24	JADE2	5	134552090	Silent	SNP	G	A
24	JKAMP	14	59487702	Missense_Mutation	SNP	C	A
24	KIAA0825	5	94484830	Frame_Shift_Del	DEL	T	-
24	KRT36	17	41487394	Missense_Mutation	SNP	G	A
24	LAMB4	7	108098437	Silent	SNP	G	A
24	LAMB4	7	108098503	Silent	SNP	G	A
24	LAMB4	7	108103219	Silent	SNP	A	G
24	LCP2	5	170258159	Silent	SNP	C	T
24	LMTK2	7	98192803	Missense_Mutation	SNP	T	A
24	MAST1	19	12852329	Splice_Region	SNP	T	C
24	METTL27	7	73834835	Missense_Mutation	SNP	T	A
24	MKRN1	7	140459921	Silent	SNP	T	C
24	MST1	3	49688595	Splice_Region	SNP	T	C
24	MT-CYB	MT	14766	Missense_Mutation	SNP	C	T
24	MUC3A	7	100958796	Silent	SNP	C	G
24	MYH6	14	23400784	Silent	SNP	G	A
24	MYO15A	17	18151139	Silent	SNP	G	A
24	NAA38	17	7857079	Silent	SNP	G	A
24	NBPF12	1	146989704	Missense_Mutation	SNP	G	A
24	NBPF3	1	21480174	Missense_Mutation	SNP	C	G
24	NDST1	5	150527971	Silent	SNP	T	G
24	NME8	7	37857294	Silent	SNP	G	A
24	NPY	7	24289514	Silent	SNP	C	T
24	NRCAM	7	108184445	Silent	SNP	C	G
24	NUDT12	5	103559276	Silent	SNP	T	C
24	NYAP1	7	100491017	Silent	SNP	C	G
24	OR2F1	7	143960380	Missense_Mutation	SNP	A	G
24	OR6C3	12	55331906	Missense_Mutation	SNP	C	T
24	OTOG	11	17645892	Missense_Mutation	SNP	G	C
24	PABPC1	8	100706669	Silent	SNP	T	C
24	PAPOLB	7	4860464	Silent	SNP	A	G
24	PAPOLB	7	4860710	Silent	SNP	G	A
24	PCYOX1	2	70261338	Missense_Mutation	SNP	C	T
24	PGM3	6	83172021	Silent	SNP	T	C
24	PHIP	6	78946845	Silent	SNP	A	T

24	PHIP	6	78946853	Silent	SNP	G	A
24	PIDD1	11	803324	Frame_Shift_Ins	INS	-	CCCTATC
24	PIDD1	11	803327	Missense_Mutation	SNP	G	C
24	PILRB	7	100356770	Missense_Mutation	SNP	G	A
24	PIMREG	17	6450359	Splice_Region	SNP	A	C
24	PKD1L1	7	47929330	Missense_Mutation	SNP	C	A
24	PLXNA4	7	132133091	Silent	SNP	T	C
24	PMPCB	7	103307593	Splice_Region	SNP	T	C
24	PRAMEF4	1	12883217	Missense_Mutation	SNP	A	C
24	PSMB6	17	4797724	Silent	SNP	C	T
24	RBAK	7	5064728	Silent	SNP	G	A
24	RBAK	7	5065502	Silent	SNP	C	T
24	RGS8	1	182671729	Missense_Mutation	SNP	T	C
24	SAMD9L	7	93132944	Missense_Mutation	SNP	G	C
24	SCIMP	17	5223379	Silent	SNP	A	G
24	SDK1	7	4174308	Silent	SNP	T	C
24	SEMA3D	7	85097924	Missense_Mutation	SNP	A	G
24	SENP6	6	75634715	Missense_Mutation	SNP	C	T
24	SERPINA5	14	94592125	Silent	SNP	A	C
24	SF3B5	6	144095487	Missense_Mutation	SNP	C	A
24	SLC12A9	7	100861173	Silent	SNP	T	C
24	SLC14A1	18	45739554	Missense_Mutation	SNP	G	A
24	SLC14A2	18	45667036	Silent	SNP	C	G
24	SLC2A4	17	7284096	Splice_Region	SNP	G	A
24	SLC34A3	9	137233633	Splice_Region	SNP	T	C
24	SPINK5	5	148120053	Silent	SNP	C	T
24	SPINK5	5	148118980	Splice_Region	SNP	C	T
24	SPINK6	5	148213934	Missense_Mutation	SNP	C	A
24	SSMEM1	7	130215997	Missense_Mutation	SNP	C	T
24	STAG3	7	100180604	Missense_Mutation	SNP	G	T
24	STK10	5	172054602	Silent	SNP	A	G
24	SVIL	10	29465715	Missense_Mutation	SNP	T	C
24	SVOPL	7	138679022	Silent	SNP	A	G
24	TANGO6	16	68860196	Missense_Mutation	SNP	C	A
24	TCF7L1	2	85309384	Silent	SNP	C	T
24	TCOF1	5	150376181	Missense_Mutation	SNP	G	C
24	TCTA	3	49412621	Silent	SNP	C	A

24	THBS4	5	80055912	Silent	SNP	C	T
24	THSD7A	7	11481915	Silent	SNP	T	C
24	TMPRSS9	19	2398851	Silent	SNP	A	G
24	TNS3	7	47396838	Missense_Mutation	SNP	C	G
24	TREML4	6	41228792	Missense_Mutation	SNP	C	T
24	TRIP6	7	100870662	Silent	SNP	A	G
24	TRPC7	5	136356887	Silent	SNP	G	A
24	TRRAP	7	98908818	Silent	SNP	C	T
24	TSHZ1	18	75286931	Silent	SNP	T	C
24	TTK	6	80031526	Silent	SNP	A	T
24	TUBB1	20	59022915	Missense_Mutation	DNP	AG	CC
24	UBQLN1	9	83680016	Missense_Mutation	SNP	C	A
24	UNC5C	4	95169261	Silent	SNP	C	A
24	USP6	17	5155513	Missense_Mutation	SNP	G	A
24	VCAN	5	83545657	Splice_Region	SNP	T	C
24	VWDE	7	12361211	Missense_Mutation	SNP	G	A
24	VWDE	7	12369637	Missense_Mutation	SNP	C	A
24	VWDE	7	12369701	Missense_Mutation	SNP	C	T
24	VWDE	7	12369896	Missense_Mutation	SNP	A	G
24	VWDE	7	12377781	Missense_Mutation	SNP	C	T
24	VWDE	7	12379482	Missense_Mutation	SNP	A	T
24	VWDE	7	12369909	Silent	SNP	A	G
24	ZAN	7	100797381	Missense_Mutation	SNP	G	C
24	ZC3HAV1	7	139055291	Missense_Mutation	SNP	G	C
24	ZC3HAV1	7	139047711	Silent	SNP	G	A
24	ZC3HAV1L	7	139028730	Missense_Mutation	SNP	C	T
24	ZNF117	7	64979235	Missense_Mutation	SNP	T	G
24	ZNF285	19	44386535	Silent	SNP	G	A
24	ZNF407	18	74635526	Silent	SNP	T	C
24	ZNF888	19	52907575	Silent	SNP	T	C
24	ZYG11A	1	52854627	Missense_Mutation	SNP	C	A
25	AC027612.2	2	91699935	Splice_Region	SNP	G	A
25	ACAP1	17	7346912	Frame_Shift_Ins	INS	-	C
25	ADCYAP1R1	7	31084842	Splice_Region	SNP	C	A
25	AGAP9	10	47502743	Silent	SNP	A	G
25	ANAPC1P2	2	87075919	Splice_Region	SNP	C	A
25	ASXL3	18	33738911	Missense_Mutation	SNP	G	A

25	BAHCC1		17	81461878	Silent	SNP	C	A
25	CCT5		5	10262551	Missense_Mutation	SNP	G	A
25	FLT3		13	28034088	In_Frame_Ins	INS	-	TAAATTTTCTCTTGG AAACTCCCATTTGAG ATCATATTCATATTCT CTGAAATCAACGTA
25	GATA2		3	128485998	Frame_Shift_Ins	INS	-	C
25	GHRH		20	37254204	Splice_Region	SNP	C	A
25	GLS2		12	56472740	Silent	SNP	C	A
25	GOLGA6L7		15	28847111	Missense_Mutation	SNP	C	T
25	IFNL2		19	39269795	Missense_Mutation	SNP	T	C
25	IQSEC2	X		53254820	Missense_Mutation	SNP	G	A
25	KLF17		1	44129375	Missense_Mutation	SNP	T	A
25	LRRC37BP1		17	30633994	Splice_Region	SNP	C	A
25	LTF		3	46439365	Silent	SNP	G	C
25	MAST4		5	67134681	Silent	SNP	C	A
25	MPPE1		18	11886587	Missense_Mutation	SNP	C	A
25	MT-CO1	MT		6251	Silent	SNP	T	C
25	MT-CO3	MT		9544	Missense_Mutation	SNP	G	A
25	MT-ND1	MT		3918	Silent	SNP	G	A
25	MT-ND5	MT		12727	Silent	SNP	T	C
25	MYH7B		20	34990810	Missense_Mutation	SNP	G	A
25	NPM1		5	171410541	Frame_Shift_Ins	INS	-	TGCT
25	NUP160		11	47840517	Missense_Mutation	SNP	C	A
25	NUP214		9	131198106	Frame_Shift_Del	DEL	A	-
25	OLFML2A		9	124810223	Silent	SNP	G	A
25	OR2T8		1	247921552	Missense_Mutation	SNP	A	G
25	OR2T8		1	247921607	Missense_Mutation	SNP	T	G
25	PARP6		15	72258036	Splice_Site	SNP	C	A
25	PCDH7		4	30723817	Missense_Mutation	SNP	G	T
25	PCDHB7		5	141174559	Missense_Mutation	SNP	C	T
25	PCDHB7		5	141174561	Missense_Mutation	SNP	T	G
25	PLXNB3	X		153768347	Missense_Mutation	DNP	GC	TG

25	PNPLA1		6	36302353	Missense_Mutation	SNP	C	A
25	POP5		12	120581116	Splice_Region	SNP	C	A
25	PRTG		15	55740620	Silent	SNP	C	T
25	PSD3		8	18872456	Silent	SNP	T	C
25	PTPN7		1	202160589	Missense_Mutation	SNP	C	A
25	PTPRG		3	62203336	Missense_Mutation	SNP	C	T
25	REXO1L8P		8	85644288	Splice_Region	SNP	T	C
25	SCRN3		2	174404153	Missense_Mutation	SNP	C	T
25	SULT1A2		16	28595911	Missense_Mutation	SNP	A	G
25	SUSD2		22	24183251	Missense_Mutation	SNP	G	A
25	TLR7	X		12886394	Missense_Mutation	SNP	C	T
25	TPSAB1		16	1242146	Missense_Mutation	SNP	G	A
25	TRIM72		16	31224312	Missense_Mutation	SNP	G	A
25	ZNF516		18	76441615	Silent	SNP	G	A
25	ZNF668		16	31061551	Silent	SNP	C	T
26	A2ML1		12	8857224	Missense_Mutation	SNP	G	A
26	AASS		7	122101420	Missense_Mutation	SNP	C	T
26	ABAT		16	8774919	Silent	SNP	C	A
26	ABCA1		9	104806424	Silent	SNP	C	T
26	ABCA5		17	69294699	Missense_Mutation	SNP	T	C
26	ABCD4		14	74290302	Missense_Mutation	SNP	G	A
26	ABHD11		7	73737314	Silent	SNP	A	G
26	ABHD16B		20	63861879	Silent	SNP	C	T
26	AC005301.1		22	16691975	Splice_Region	SNP	G	A
26	AC008397.2		19	18218974	Missense_Mutation	SNP	C	T
26	AC024592.3		19	5867737	Missense_Mutation	SNP	G	T
26	ACE		17	63489064	Missense_Mutation	SNP	G	A
26	ACKR1		1	159205737	Missense_Mutation	SNP	G	A
26	ACOX3		4	8410281	Silent	SNP	G	A
26	ACRBP		12	6638412	Splice_Region	SNP	G	C
26	ACSF3		16	89114475	Missense_Mutation	SNP	G	A
26	ACVRL1		12	51915395	Missense_Mutation	SNP	G	A
26	ADAD2		16	84191296	Silent	SNP	G	A
26	ADAD2		16	84195974	Silent	SNP	C	T
26	ADAMTS14		10	70753879	Missense_Mutation	SNP	C	A
26	ADAMTS14		10	70753926	Silent	SNP	C	T
26	ADAMTS14		10	70760574	Silent	SNP	A	G

26	ADAMTS18	16	77326022	Missense_Mutation	SNP	A	T
26	ADAMTS4	1	161198214	Silent	SNP	C	T
26	ADAMTS7	15	78771738	Silent	SNP	G	A
26	ADAMTSL2	9	133573909	Missense_Mutation	SNP	G	A
26	ADCY9	16	4114363	Silent	SNP	C	T
26	ADGRB3	6	69361281	Silent	SNP	G	A
26	ADGRD1	12	130987154	Missense_Mutation	SNP	G	A
26	ADGRF2	6	47656563	Missense_Mutation	SNP	A	G
26	ADGRF2	6	47681837	Missense_Mutation	DNP	CA	TG
26	ADGRV1	5	90683772	Missense_Mutation	SNP	G	A
26	ADGRV1	5	90694507	Missense_Mutation	SNP	A	G
26	ADRA1B	5	159917454	Silent	SNP	G	A
26	ADSS1	14	104740703	Silent	SNP	C	T
26	AGAP2	12	57732908	Missense_Mutation	SNP	G	A
26	AGGF1	5	77063199	Missense_Mutation	SNP	C	A
26	AGO2	8	140556212	Silent	SNP	C	T
26	AHNAK	11	62526476	Silent	SNP	G	A
26	AHNAK2	14	104945922	Missense_Mutation	DNP	GG	CC
26	AHNAK2	14	104949132	Missense_Mutation	SNP	T	C
26	AHNAK2	14	104949271	Missense_Mutation	SNP	G	C
26	AJM1	9	136845620	Silent	SNP	G	A
26	AK9	6	109614403	Missense_Mutation	SNP	A	G
26	AKAP6	14	32823537	Silent	SNP	A	C
26	ALPK2	18	58535842	Missense_Mutation	SNP	G	A
26	ALX4	11	44265016	Silent	SNP	G	A
26	ANGEL1	14	76809237	Missense_Mutation	SNP	C	A
26	ANKRD34C	15	79294478	Silent	SNP	C	T
26	ANKRD62	18	12096250	Missense_Mutation	SNP	G	T
26	ANO8	19	17330215	Missense_Mutation	SNP	C	T
26	ANO9	11	429659	Splice_Region	SNP	C	T
26	ANTXRL	10	46311575	Silent	SNP	G	A
26	AOAH	7	36724083	Silent	SNP	C	T
26	AOC3	17	42851819	Missense_Mutation	SNP	G	A
26	AP002748.5	11	66514624	Silent	SNP	G	A
26	ARHGEF10L	1	17632381	Missense_Mutation	SNP	G	A
26	ARHGEF10L	1	17637979	Silent	SNP	C	T
26	ARHGEF12	11	120451607	Missense_Mutation	SNP	C	T

26	ARHGEF18		19	7468881	Silent	SNP	T	G
26	ARHGEF37		5	149627163	Missense_Mutation	SNP	A	C
26	ARMCX2	X		101656080	Silent	SNP	T	C
26	ASAP3		1	23442255	Missense_Mutation	SNP	C	T
26	ASXL1		20	32433908	Silent	SNP	G	A
26	ASXL3		18	33743197	Nonsense_Mutation	SNP	C	T
26	ATP2C2		16	84460669	Silent	SNP	C	T
26	AXIN1		16	346164	Missense_Mutation	SNP	C	T
26	BAHCC1		17	81442460	Missense_Mutation	SNP	G	A
26	BCAS1		20	53944930	Missense_Mutation	SNP	A	G
26	BCAS1		20	54058649	Missense_Mutation	SNP	G	T
26	BCL9L		11	118898733	Silent	SNP	A	G
26	BCORL1	X		130021129	Nonsense_Mutation	SNP	C	T
26	BMS1		10	42796958	Missense_Mutation	SNP	G	A
26	BNIP2		15	59689292	Missense_Mutation	SNP	T	G
26	BPIFB3		20	33064486	Missense_Mutation	SNP	G	A
26	BRD1		22	49823676	Silent	SNP	G	A
26	BRINP3		1	190160745	Silent	SNP	C	T
26	BSN		3	49656880	Missense_Mutation	SNP	C	T
26	C16orf78		16	49396479	Missense_Mutation	SNP	C	T
26	C1QTNF2		5	160349630	Silent	SNP	C	T
26	C2CD2L		11	119114318	Missense_Mutation	SNP	C	T
26	C3		19	6702146	Silent	SNP	C	G
26	C3orf22		3	126553307	Silent	SNP	C	T
26	C3orf49		3	63831754	Missense_Mutation	SNP	G	C
26	C4BPB		1	207090390	Silent	SNP	C	T
26	C4orf54		4	99650605	Silent	SNP	G	A
26	C6orf118		6	165301974	Silent	SNP	C	T
26	CA6		1	8945916	Silent	SNP	G	A
26	CABLES1		18	23253786	Silent	SNP	G	A
26	CACNA1F	X		49233305	Missense_Mutation	SNP	G	A
26	CACNA1G		17	50599555	Missense_Mutation	SNP	G	A
26	CAMKK2		12	121253293	Missense_Mutation	SNP	G	A
26	CAPN15		16	552313	Silent	SNP	C	A
26	CAPN9		1	230762748	Silent	SNP	C	T
26	CARD11		7	2930046	Silent	SNP	G	A
26	CARMIL2		16	67649546	Missense_Mutation	SNP	A	G

26	CCDC102B	18	68837114	Silent	SNP	G	A
26	CCDC112	5	115271374	Missense_Mutation	SNP	G	A
26	CCDC138	2	108791752	Missense_Mutation	SNP	G	A
26	CCDC175	14	59538126	Missense_Mutation	SNP	C	T
26	CCDC63	12	110904754	Silent	SNP	G	T
26	CCDC68	18	54937957	Splice_Region	SNP	C	T
26	CCDC71	3	49163259	Missense_Mutation	SNP	T	A
26	CCDC77	12	438517	Missense_Mutation	SNP	T	C
26	CCDC81	11	86375179	Missense_Mutation	SNP	G	A
26	CCIN	9	36169726	Missense_Mutation	SNP	G	A
26	CCNY	10	35530182	Missense_Mutation	SNP	G	A
26	CDH1	16	68812262	Missense_Mutation	SNP	C	T
26	CDH12	5	22078475	Missense_Mutation	SNP	C	T
26	CDH3	16	68698202	Silent	SNP	G	A
26	CDH5	16	66402732	Missense_Mutation	SNP	G	A
26	CDR2L	17	75003966	Silent	SNP	A	G
26	CEACAM20	19	44520547	Silent	SNP	G	C
26	CELSR2	1	109258576	Missense_Mutation	SNP	C	T
26	CENPE	4	103145304	Missense_Mutation	SNP	A	G
26	CENPE	4	103161192	Silent	SNP	C	T
26	CFAP94	12	25158555	Missense_Mutation	SNP	G	T
26	CFL1	11	65856048	Silent	SNP	G	A
26	CHDH	3	53823776	Missense_Mutation	SNP	A	C
26	CHRD	3	184386521	Silent	SNP	C	T
26	CLCC1	1	108943574	Silent	SNP	A	G
26	CLEC12B	12	10010776	Missense_Mutation	SNP	C	A
26	CLEC12B	12	10014678	Missense_Mutation	SNP	G	C
26	CLEC12B	12	10010780	Silent	SNP	C	T
26	CLEC12B	12	10012870	Silent	SNP	A	G
26	CLSTN2	3	140558820	Silent	SNP	C	T
26	CNIH2	11	66283241	Splice_Region	SNP	G	A
26	CNR2	1	23874619	Silent	SNP	C	T
26	COBL	7	51028450	Silent	SNP	A	G
26	COBLL1	2	164694691	Missense_Mutation	SNP	T	C
26	COL11A1	1	102886895	Silent	SNP	A	G
26	COL4A4	2	227007466	Silent	SNP	G	A
26	COL4A4	2	227032170	Silent	SNP	C	T

26	COL6A5	3	130406272	Missense_Mutation	SNP	G	C
26	COL6A5	3	130406145	Silent	SNP	C	T
26	COL7A1	3	48587045	Missense_Mutation	SNP	C	T
26	CPAMD8	19	16977489	Missense_Mutation	SNP	T	C
26	CPNE7	16	89595545	Missense_Mutation	SNP	G	A
26	CRACD	4	56316592	Silent	SNP	C	T
26	CREBBP	16	3729448	Missense_Mutation	SNP	G	A
26	CRELD1	3	9943972	Silent	SNP	C	T
26	CRELD1	3	9938107	Splice_Site	SNP	G	A
26	CRIM1	2	36479645	Silent	SNP	C	G
26	CRYBG1	6	106512086	Silent	SNP	A	G
26	CSAG1	X	152727846	Missense_Mutation	SNP	C	T
26	CSPP1	8	67164469	Missense_Mutation	SNP	G	A
26	CST11	20	23452712	Missense_Mutation	SNP	C	T
26	CTNNA1	5	138930857	Silent	SNP	G	A
26	CTPS1	1	40984895	Missense_Mutation	SNP	C	T
26	CYP2S1	19	41198802	Missense_Mutation	SNP	C	T
26	DAAM1	14	59355329	Missense_Mutation	SNP	G	A
26	DAZAP1	19	1429991	Missense_Mutation	SNP	C	T
26	DCAF4	14	72955642	Silent	SNP	T	G
26	DCAF8L2	X	27747928	Missense_Mutation	SNP	A	G
26	DCHS2	4	154366383	Missense_Mutation	SNP	T	C
26	DEAF1	11	691532	Missense_Mutation	SNP	G	A
26	DHFR2	3	94061265	Missense_Mutation	SNP	G	C
26	DIPK1B	9	136723174	Silent	SNP	G	A
26	DMBT1	10	122631205	Silent	SNP	T	C
26	DNAH11	7	21559615	Silent	SNP	C	T
26	DNAH9	17	11689853	Missense_Mutation	SNP	G	A
26	DNMT3A	2	25244214	Nonsense_Mutation	SNP	G	A
26	DPEP1	16	89637957	Missense_Mutation	SNP	G	C
26	DVL2	17	7229894	Silent	SNP	G	A
26	DYNC1H1	14	102038572	Missense_Mutation	SNP	C	T
26	DZIP1L	3	138097804	Missense_Mutation	SNP	C	T
26	EAPP	14	34516439	Silent	SNP	G	A
26	ECT2L	6	138838419	Missense_Mutation	SNP	C	T
26	EFCAB12	3	129401728	Silent	SNP	C	T
26	EFHD1	2	232633959	Silent	SNP	G	T

26	EFTUD2	17	44854626	Missense_Mutation	SNP	C	T
26	EIF2D	1	206595795	Missense_Mutation	SNP	C	T
26	EIF2S3B	12	10507052	Missense_Mutation	SNP	G	A
26	EIF4G1	3	184327898	Silent	SNP	C	T
26	ELAVL3	19	11466233	Missense_Mutation	SNP	T	C
26	ELFN2	22	37375118	Silent	SNP	G	A
26	EN2	7	155462637	Silent	SNP	T	C
26	ENO3	17	4953081	Missense_Mutation	SNP	A	G
26	ENO3	17	4953285	Missense_Mutation	SNP	T	C
26	EPAS1	2	46382510	Silent	SNP	C	T
26	EPHA10	1	37761720	Missense_Mutation	SNP	C	T
26	EPHA7	6	93255925	Missense_Mutation	SNP	C	T
26	ERVV-2	19	53050433	Silent	SNP	G	A
26	ESCO1	18	21568029	Silent	SNP	C	T
26	ESPL1	12	53268840	Missense_Mutation	SNP	C	A
26	EVC2	4	5568494	Silent	SNP	G	A
26	EXD2	14	69236441	Silent	SNP	A	G
26	EXD3	9	137351100	Missense_Mutation	SNP	C	T
26	EXT1	8	117807339	Silent	SNP	C	T
26	EYS	6	65295964	Missense_Mutation	SNP	T	A
26	F13B	1	197057327	Missense_Mutation	SNP	C	T
26	F8	X	154930010	Missense_Mutation	SNP	G	C
26	FAM126A	7	22976212	Splice_Region	SNP	T	C
26	FAM187B	19	35228354	Silent	SNP	G	A
26	FAM214A	15	52609086	Missense_Mutation	DNP	TT	CC
26	FAM50B	6	3850082	Missense_Mutation	SNP	C	T
26	FAM71F2	7	128675828	Missense_Mutation	SNP	C	A
26	FAM71F2	7	128675737	Silent	SNP	C	G
26	FAM83C	20	35287168	Silent	SNP	G	A
26	FANCA	16	89783072	Missense_Mutation	SNP	C	T
26	FANCM	14	45176512	Missense_Mutation	SNP	A	G
26	FAR2	12	29270527	Silent	SNP	G	A
26	FBH1	10	5909062	Splice_Region	SNP	T	C
26	FEM1A	19	4792925	Silent	SNP	C	T
26	FLG	1	152305078	Missense_Mutation	SNP	G	A
26	FLT1	13	28319505	Silent	SNP	A	G
26	FNDC1	6	159232512	Missense_Mutation	SNP	G	A

26	FRAS1	4	78413006	Missense_Mutation	SNP	G	T
26	FSD1	19	4306314	Silent	SNP	T	C
26	FZD9	7	73435473	Missense_Mutation	SNP	C	T
26	GABRA4	4	46993349	Missense_Mutation	SNP	G	T
26	GALNT14	2	30912318	Missense_Mutation	SNP	G	T
26	GBP3	1	89007770	Missense_Mutation	SNP	A	G
26	GCNT3	15	59619222	Silent	SNP	A	T
26	GET3	19	12748034	Missense_Mutation	SNP	G	A
26	GET3	19	12748048	Missense_Mutation	SNP	G	C
26	GGN	19	38385946	Missense_Mutation	SNP	G	T
26	GLRX3	10	130169429	Splice_Region	SNP	T	A
26	GNL3	3	52693241	Missense_Mutation	SNP	G	A
26	GPBP1	5	57230956	Silent	SNP	G	A
26	GPR108	19	6731046	Silent	SNP	T	C
26	GPSM2	1	108885572	Missense_Mutation	SNP	G	A
26	GRK1	13	113667974	Silent	SNP	A	G
26	GSDMB	17	39905943	Missense_Mutation	SNP	G	A
26	GSDME	7	24717254	Missense_Mutation	SNP	C	T
26	GSK3B	3	119947347	Missense_Mutation	SNP	C	T
26	GSTA1	6	52794164	Silent	SNP	T	C
26	GUCY2C	12	14688001	Missense_Mutation	SNP	C	T
26	GUCY2D	17	8006623	Silent	SNP	C	T
26	GYPC	2	126696088	Silent	SNP	A	C
26	H4C8	6	26285332	Silent	SNP	A	G
26	HASPIN	17	3724546	Missense_Mutation	SNP	G	A
26	HCAR3	12	122716221	Missense_Mutation	SNP	T	G
26	HECTD4	12	112184610	Silent	SNP	G	A
26	HERC2	15	28238663	Silent	SNP	G	A
26	HERPUD1	16	56935236	Missense_Mutation	SNP	G	A
26	HEXB	5	74689390	Missense_Mutation	SNP	A	G
26	HIP1	7	75559829	Silent	SNP	C	T
26	HIVEP2	6	142760268	Missense_Mutation	SNP	G	A
26	HMCN2	9	130430524	Missense_Mutation	SNP	G	A
26	HNF1A	12	120988986	Silent	SNP	C	T
26	HS3ST4	16	26136133	Missense_Mutation	SNP	G	A
26	HSD3B1	1	119514473	Missense_Mutation	SNP	G	A
26	HSDL1	16	84124643	Missense_Mutation	SNP	G	C

26	HSP90B1	12	103934201	Silent	SNP	C	T
26	HSPG2	1	21836897	Silent	SNP	G	A
26	HTR1B	6	77463033	Missense_Mutation	SNP	A	C
26	HYAL4	7	123868721	Nonsense_Mutation	SNP	C	T
26	IDH1	2	208248389	Missense_Mutation	SNP	G	A
26	IDUA	4	1000664	Silent	SNP	C	T
26	IFIT2	10	89306096	Missense_Mutation	SNP	G	A
26	IFIT2	10	89306703	Silent	SNP	T	A
26	IFIT5	10	89418243	Silent	SNP	C	T
26	IFITM3	11	320805	Missense_Mutation	SNP	G	T
26	IGLV6-57	22	22196050	Missense_Mutation	SNP	G	C
26	IGSF22	11	18709457	Silent	SNP	C	G
26	IKZF3	17	39766006	Silent	SNP	G	A
26	IL1B	2	112832813	Silent	SNP	G	A
26	IL27RA	19	14050824	Missense_Mutation	SNP	C	T
26	IMPDH1	7	128394575	Silent	SNP	C	T
26	INPP5J	22	31125418	Missense_Mutation	SNP	G	A
26	ITGA2B	17	44372421	Splice_Region	SNP	G	A
26	ITGA3	17	50068131	Missense_Mutation	SNP	G	A
26	ITGAE	17	3729542	Missense_Mutation	SNP	G	A
26	ITIH4	3	52818044	Splice_Region	SNP	T	C
26	ITIH5	10	7576723	Missense_Mutation	SNP	T	G
26	ITIH5	10	7576538	Silent	SNP	A	G
26	JAKMIP3	10	132140507	Silent	SNP	C	T
26	JPH2	20	44118607	Missense_Mutation	SNP	C	T
26	JPH3	16	87690232	Silent	SNP	T	C
26	KCNB2	8	72936223	Missense_Mutation	SNP	G	A
26	KCNG4	16	84222497	Missense_Mutation	SNP	C	T
26	KCNH2	7	150947340	Missense_Mutation	SNP	C	A
26	KCNH4	17	42170234	Silent	SNP	T	C
26	KCNJ12	17	21415351	Silent	SNP	G	A
26	KCNQ2	20	63408523	Missense_Mutation	SNP	C	T
26	KDM2B	12	121442717	Silent	SNP	G	A
26	KDM5A	12	331912	Silent	SNP	G	A
26	KDM6B	17	7851675	Silent	SNP	G	A
26	KIF1B	1	10337085	Silent	SNP	G	A
26	KIF26A	14	104177084	Silent	SNP	T	C

26	KIF26A		14	104178692	Silent	SNP	C	T
26	KLHL29		2	23642627	Silent	SNP	C	T
26	KLHL31		6	53654264	Silent	SNP	A	G
26	KLHL36		16	84661674	Silent	SNP	A	G
26	KLRF2		12	9888765	Missense_Mutation	SNP	G	A
26	KMT2A		11	118502524	Missense_Mutation	SNP	G	A
26	KRBA1		7	149720923	Silent	SNP	C	T
26	KRT73		12	52618344	Missense_Mutation	SNP	C	T
26	KRT82		12	52401326	Missense_Mutation	SNP	C	T
26	KRTAP10-4		21	44573789	Missense_Mutation	SNP	A	G
26	KRTAP10-9		21	44627716	Missense_Mutation	SNP	A	G
26	KRTAP10-9		21	44627940	Missense_Mutation	SNP	C	T
26	LAMA5		20	62338048	Missense_Mutation	SNP	C	T
26	LDLR		19	11116124	Silent	SNP	C	T
26	LDLRAD1		1	54009054	Silent	SNP	C	T
26	LIPC		15	58541793	Silent	SNP	C	T
26	LMBRD2		5	36142546	Silent	SNP	A	G
26	LRP2		2	169257123	Splice_Site	SNP	C	T
26	LRP5		11	68357684	Missense_Mutation	SNP	C	T
26	LRPAP1		4	3525059	Splice_Region	SNP	C	T
26	LRRC14		8	144519870	Missense_Mutation	SNP	G	A
26	LRRC59		17	50397297	Missense_Mutation	SNP	C	A
26	MADD		11	47276809	Silent	SNP	G	A
26	MAGEB6B	X		26161415	Missense_Mutation	SNP	A	G
26	MAN2B2		4	6621284	Silent	SNP	G	A
26	MAOA	X		43744144	Silent	SNP	T	C
26	MAP1A		15	43522919	Silent	SNP	A	G
26	MAP3K6		1	27362142	Missense_Mutation	SNP	G	A
26	MAPK9		5	180249062	Missense_Mutation	SNP	G	A
26	MASP1		3	187221124	Missense_Mutation	SNP	G	A
26	MAST2		1	46035164	Missense_Mutation	SNP	C	T
26	MATK		19	3779400	Missense_Mutation	SNP	C	T
26	MEN1		11	64805130	Silent	SNP	G	A
26	METTL2B		7	128498098	Missense_Mutation	SNP	G	A
26	MFHAS1		8	8891716	Missense_Mutation	SNP	G	T
26	MGAT4B		5	179801576	Silent	SNP	G	A
26	MMP2		16	55493201	Silent	SNP	G	A

26	MMP27		11	102702838	Silent	SNP	C	A
26	MMS19		10	97460109	Missense_Mutation	SNP	G	A
26	MRGPRX1		11	18934647	Silent	SNP	T	C
26	MRGPRX4		11	18173801	Missense_Mutation	SNP	C	T
26	MS4A12		11	60497529	Nonsense_Mutation	SNP	C	T
26	MS4A14		11	60416718	Missense_Mutation	SNP	G	A
26	MS4A5		11	60430855	Silent	SNP	C	T
26	MS4A7		11	60385111	Silent	SNP	G	A
26	MTA1		14	105464756	Missense_Mutation	SNP	C	T
26	MTCH1		6	36969864	Missense_Mutation	SNP	C	T
26	MTF1		1	37840122	Missense_Mutation	SNP	G	A
26	MTHFR		1	11796321	Missense_Mutation	SNP	G	A
26	MTMR3		22	30008007	Silent	SNP	C	T
26	MUC16		19	8937666	Missense_Mutation	SNP	C	T
26	MUC16		19	8946306	Missense_Mutation	SNP	G	A
26	MUC16		19	8948231	Missense_Mutation	SNP	G	C
26	MUC16		19	8953002	Missense_Mutation	SNP	T	G
26	MUC16		19	8964274	Missense_Mutation	SNP	G	T
26	MUC16		19	8965293	Missense_Mutation	SNP	C	T
26	MUC16		19	8965407	Missense_Mutation	SNP	G	A
26	MUC16		19	8966052	Missense_Mutation	SNP	C	T
26	MUC16		19	8964908	Silent	SNP	T	A
26	MUC16		19	8966588	Silent	SNP	T	G
26	MUC5B		11	1243735	Silent	SNP	G	A
26	MXRA5	X		3323009	Silent	SNP	G	A
26	MYCBP2		13	77098931	Silent	SNP	C	T
26	MYEOV		11	69296009	Missense_Mutation	SNP	C	T
26	MYH7		14	23427840	Missense_Mutation	SNP	C	T
26	MYOZ1		10	73634614	Silent	SNP	G	A
26	NADSYN1		11	71491897	Silent	SNP	C	T
26	NAGK		2	71070565	Silent	SNP	T	C
26	NANS		9	98060802	Silent	SNP	T	C
26	NAP1L1		12	76060186	Silent	SNP	G	A
26	NAP1L3	X		93672635	Missense_Mutation	SNP	G	C
26	NAV2		11	20083123	Silent	SNP	G	A
26	NBEAL1		2	203126066	Silent	SNP	T	G
26	NBEAL2		3	46995266	Missense_Mutation	SNP	C	G

26	NCOA5	20	46063562	Missense_Mutation	SNP	C	T
26	NCOR2	12	124354174	Silent	SNP	C	T
26	NDNF	4	121037032	Silent	SNP	A	G
26	NEDD9	6	11193627	Silent	SNP	G	A
26	NEDD9	6	11213599	Silent	SNP	T	C
26	NEK10	3	27352486	Silent	SNP	G	A
26	NEK11	3	131228591	Missense_Mutation	SNP	A	T
26	NEMF	14	49840770	Missense_Mutation	SNP	G	A
26	NFATC1	18	79486406	Missense_Mutation	SNP	T	G
26	NINL	20	25476252	Silent	SNP	A	G
26	NKX2-5	5	173235021	Silent	SNP	T	C
26	NLRC5	16	57026812	Silent	SNP	G	A
26	NLRP11	19	55817968	Silent	SNP	G	C
26	NME4	16	399659	Silent	SNP	G	A
26	NME4	16	400309	Silent	SNP	C	T
26	NOTCH1	9	136522979	Missense_Mutation	SNP	C	T
26	NOTCH1	9	136510809	Splice_Region	SNP	C	T
26	NPAS4	11	66421290	Silent	SNP	G	T
26	NPVF	7	25226781	Silent	SNP	G	A
26	NR2E3	15	71813543	Missense_Mutation	SNP	G	A
26	NT5DC3	12	103793168	Silent	SNP	G	T
26	NTNG2	9	132198490	Silent	SNP	C	T
26	NUDT17	1	145846617	Missense_Mutation	SNP	G	A
26	NUP210	3	13319244	Missense_Mutation	SNP	G	A
26	OBSCN	1	228308313	Silent	SNP	G	A
26	OCA2	15	27851392	Silent	SNP	A	G
26	ODF4	17	8340280	Missense_Mutation	SNP	C	T
26	OLIG2	21	33027093	Silent	SNP	A	G
26	OPLAH	8	144054725	Missense_Mutation	SNP	G	A
26	OR1I1	19	15087795	Missense_Mutation	SNP	C	T
26	OR1I1	19	15088040	Silent	SNP	A	T
26	OR1S2	11	58204006	Missense_Mutation	SNP	A	G
26	OR2G2	1	247588717	Missense_Mutation	SNP	G	C
26	OR4C15	11	55554946	Missense_Mutation	SNP	C	A
26	OR4N2	14	19827619	Missense_Mutation	DNP	CC	AG
26	OR4N2	14	19827604	Silent	SNP	C	T
26	OR51D1	11	4640521	Missense_Mutation	SNP	G	A

26	OR51S1	11	4849031	Missense_Mutation	SNP	G	C
26	OR52L1	11	5986669	Missense_Mutation	SNP	C	T
26	OR52N1	11	5788318	Missense_Mutation	SNP	G	A
26	OR5M11	11	56542420	Missense_Mutation	SNP	C	G
26	OR6F1	1	247712113	Missense_Mutation	SNP	A	G
26	OR6F1	1	247712306	Silent	SNP	C	G
26	OR7A5	19	14827804	Silent	SNP	T	C
26	OR8S1	12	48526013	Missense_Mutation	SNP	C	T
26	OVCH1	12	29464617	Missense_Mutation	SNP	G	A
26	PABPC4L	4	134200775	Missense_Mutation	SNP	C	T
26	PARP10	8	143984371	Missense_Mutation	SNP	T	C
26	PARP10	8	143984818	Missense_Mutation	SNP	A	G
26	PARP4	13	24426479	Missense_Mutation	SNP	C	G
26	PARP4	13	24431457	Missense_Mutation	SNP	G	A
26	PARP4	13	24435159	Missense_Mutation	SNP	G	T
26	PARP4	13	24435303	Missense_Mutation	SNP	C	G
26	PBX1	1	164559883	Missense_Mutation	SNP	G	A
26	PCSK9	1	55039995	Missense_Mutation	SNP	C	T
26	PDE11A	2	177817928	Splice_Region	SNP	A	G
26	PDE6C	10	93612977	Silent	SNP	G	A
26	PDE7B	6	136038308	Silent	SNP	A	C
26	PDLIM3	4	185504577	Missense_Mutation	SNP	G	A
26	PER1	17	8147768	Missense_Mutation	SNP	C	T
26	PERP	6	138107166	Missense_Mutation	SNP	C	T
26	PEX1	7	92501913	Missense_Mutation	SNP	C	T
26	PGBD4	15	34104207	Missense_Mutation	SNP	C	T
26	PIGK	1	77219357	Missense_Mutation	SNP	T	C
26	PIGX	3	196733809	Silent	SNP	C	T
26	PIK3AP1	10	96709750	Missense_Mutation	SNP	C	A
26	PIK3CG	7	106884244	Silent	SNP	C	T
26	PIK3R1	5	68273403	Silent	SNP	G	A
26	PIKFYVE	2	208325923	Nonsense_Mutation	SNP	C	T
26	PINLYP	19	43581675	Silent	SNP	C	T
26	PKHD1	6	52026025	Missense_Mutation	SNP	G	A
26	PLCB2	15	40296801	Silent	SNP	A	G
26	PLCB3	11	64267514	Silent	SNP	G	A
26	PLCZ1	12	18736215	Splice_Region	SNP	T	C

26	PLEK	2	68386578	Silent	SNP	G	A
26	PLEKHH2	2	43700235	Missense_Mutation	SNP	A	G
26	PLIN4	19	4511128	Silent	SNP	C	G
26	PLXDC2	10	20147909	Splice_Region	SNP	G	C
26	PLXNA4	7	132508190	Silent	SNP	T	C
26	PNMA8A	19	46470724	Silent	SNP	G	A
26	PNMA8B	19	46494979	Missense_Mutation	SNP	C	T
26	POC5	5	75685328	Missense_Mutation	SNP	G	A
26	POLD3	11	74636230	Missense_Mutation	SNP	C	T
26	POLE	12	132673626	Silent	SNP	G	A
26	POLR1A	2	86032320	Silent	SNP	C	T
26	POLRMT	19	625198	Silent	SNP	A	G
26	POMT2	14	77278826	Silent	SNP	G	A
26	PPL	16	4895374	Missense_Mutation	SNP	C	T
26	PPP1R15A	19	48875737	Missense_Mutation	SNP	A	G
26	PPP3R1	2	68188635	Silent	SNP	G	A
26	PRDX3	10	119172417	Silent	SNP	G	A
26	PRKACG	9	69013291	Missense_Mutation	SNP	G	C
26	PRNP	20	4699605	Missense_Mutation	SNP	A	G
26	PROM1	4	16009071	Silent	SNP	G	A
26	PRRG4	11	32853373	Missense_Mutation	SNP	C	A
26	PRUNE2	9	76709263	Missense_Mutation	SNP	G	A
26	PRX	19	40396509	Missense_Mutation	SNP	C	T
26	PSG1	19	42871817	Missense_Mutation	SNP	C	T
26	PTCH1	9	95453610	Missense_Mutation	SNP	G	A
26	PTPN14	1	214393732	Nonsense_Mutation	SNP	G	A
26	PTPRH	19	55202167	Missense_Mutation	SNP	G	A
26	PTPRQ	12	80635059	Silent	SNP	C	T
26	PTTG2	4	37960512	Silent	SNP	G	A
26	PYGB	20	25284178	Silent	SNP	T	C
26	RAB3A	19	18200389	Silent	SNP	A	G
26	RAB3D	19	11335468	Missense_Mutation	SNP	G	A
26	RAB8B	15	63256588	Silent	SNP	G	A
26	RAD18	3	8881405	Silent	SNP	G	A
26	RAPGEF1	9	131627980	Silent	SNP	G	A
26	RAPSN	11	47447887	Silent	SNP	A	G
26	RBM34	1	235160580	Missense_Mutation	SNP	G	A

26	RC3H2	9	122855801	Silent	SNP	T	G
26	RC3H2	9	122890478	Silent	SNP	G	A
26	RELN	7	103489758	Silent	SNP	G	A
26	REV1	2	99442408	Missense_Mutation	SNP	C	T
26	RFX2	19	6047428	Silent	SNP	C	T
26	RGL4	22	23692101	Missense_Mutation	SNP	A	G
26	RGL4	22	23693783	Missense_Mutation	SNP	C	T
26	RIMBP2	12	130407734	Missense_Mutation	SNP	C	T
26	RIOX2	3	97945881	Missense_Mutation	SNP	C	T
26	RMC1	18	23518953	Silent	SNP	G	A
26	RNF25	2	218665205	Missense_Mutation	SNP	C	T
26	ROBO2	3	77550916	Silent	SNP	G	A
26	RRM2B	8	102238763	Silent	SNP	C	T
26	RSC1A1	1	15660052	Missense_Mutation	SNP	T	C
26	RYR1	19	38486002	Missense_Mutation	SNP	G	A
26	SACS	13	23335886	Missense_Mutation	SNP	C	T
26	SALL1	16	51141570	Missense_Mutation	SNP	C	T
26	SAMD11	1	944041	Missense_Mutation	SNP	C	T
26	SAP30BP	17	75704807	Missense_Mutation	SNP	G	A
26	SCP2	1	52978229	Silent	SNP	T	C
26	SCYL1	11	65538086	Silent	SNP	C	T
26	SDAD1	4	75957563	Missense_Mutation	SNP	G	C
26	SELP	1	169597075	Missense_Mutation	SNP	C	T
26	SEMA3A	7	84110533	Silent	SNP	G	A
26	SENP2	3	185613377	Missense_Mutation	SNP	C	A
26	SEPTIN12	16	4785843	Missense_Mutation	SNP	G	A
26	SFXN5	2	73040876	Missense_Mutation	SNP	C	T
26	SH2D3C	9	127749457	Missense_Mutation	SNP	C	T
26	SHROOM3	4	76741299	Silent	SNP	G	A
26	SHROOM4	X	50607674	Silent	SNP	T	C
26	SIL1	5	139121126	Silent	SNP	T	C
26	SIN3B	19	16869758	Missense_Mutation	SNP	C	T
26	SIPA1L3	19	38082171	Silent	SNP	C	T
26	SLC11A2	12	50992283	Silent	SNP	A	G
26	SLC13A5	17	6695838	Missense_Mutation	SNP	C	T
26	SLC1A6	19	14952945	Silent	SNP	A	G
26	SLC24A3	20	19585018	Silent	SNP	G	A

26	SLC24A4	14	92442163	Silent	SNP	G	A
26	SLC25A26	3	66369532	Missense_Mutation	SNP	C	T
26	SLC25A26	3	66369518	Silent	SNP	A	G
26	SLC25A47	14	100329001	Silent	SNP	G	C
26	SLC28A1	15	84904200	Missense_Mutation	SNP	G	A
26	SLC28A2	15	45262069	Missense_Mutation	SNP	C	A
26	SLC39A8	4	102304356	Silent	SNP	T	C
26	SLC6A5	11	20601477	Silent	SNP	C	T
26	SLC6A7	5	150204571	Missense_Mutation	SNP	G	A
26	SLC9A3	5	479914	Silent	SNP	C	T
26	SMG6	17	2299881	Missense_Mutation	SNP	C	G
26	SMOC2	6	168650751	Silent	SNP	C	T
26	SNW1	14	77731022	Silent	SNP	C	T
26	SNX19	11	130914859	Missense_Mutation	SNP	C	G
26	SNX29	16	12048479	Missense_Mutation	SNP	G	A
26	SOGA3	6	127476034	Silent	SNP	G	A
26	SP110	2	230207994	Missense_Mutation	SNP	C	T
26	SPEN	1	15928919	Silent	SNP	C	T
26	SPEN	1	15937835	Silent	SNP	C	G
26	SPP2	2	234058895	Silent	SNP	T	A
26	SPTA1	1	158642446	Missense_Mutation	SNP	A	G
26	SPTB	14	64769115	Missense_Mutation	SNP	G	A
26	SPTB	14	64782334	Missense_Mutation	SNP	C	G
26	STAP2	19	4333714	Missense_Mutation	SNP	C	T
26	STARD9	15	42690082	Missense_Mutation	SNP	T	C
26	STIM1	11	4082294	Silent	SNP	A	G
26	STRA6	15	74197387	Missense_Mutation	SNP	G	A
26	SULF1	8	69604925	Missense_Mutation	SNP	C	T
26	SYNE2	14	64090978	Missense_Mutation	SNP	G	A
26	SYNE2	14	64146140	Missense_Mutation	SNP	C	A
26	SYNE2	14	64146127	Silent	SNP	C	T
26	SYNE2	14	64170429	Silent	SNP	C	A
26	SYNE2	14	64139936	Splice_Region	SNP	G	C
26	SYNE3	14	95439984	Missense_Mutation	SNP	G	A
26	SYNM	15	99131590	Missense_Mutation	SNP	C	T
26	SZT2	1	43446193	Silent	SNP	G	A
26	TACC2	10	122083008	Missense_Mutation	SNP	G	A

26	TACC2	10	122086970	Silent	SNP	A	G
26	TAF4B	18	26321139	Missense_Mutation	SNP	G	A
26	TAPBPL	12	6458741	Missense_Mutation	SNP	C	T
26	TAS2R41	7	143478061	Silent	SNP	G	A
26	TBC1D26	17	15741192	Missense_Mutation	SNP	C	T
26	TBL3	16	1975205	Silent	SNP	A	C
26	TBL3	16	1975957	Splice_Region	SNP	G	A
26	TBX3	12	114679532	Silent	SNP	G	A
26	TBXT	6	166166700	Silent	SNP	G	A
26	TCP11L1	11	33058034	Missense_Mutation	SNP	A	G
26	TDRD10	1	154544001	Missense_Mutation	SNP	G	A
26	TECTB	10	112293788	Silent	SNP	C	G
26	TEK	9	27213582	Silent	SNP	T	C
26	TERT	5	1266481	Silent	SNP	G	A
26	TEX52	12	2849462	Silent	SNP	C	T
26	TG	8	133096302	Missense_Mutation	SNP	T	C
26	TGFB1	19	41341955	Missense_Mutation	SNP	G	A
26	TGIF2LX	X	89922674	Missense_Mutation	SNP	G	A
26	THSD7B	2	137618402	Silent	SNP	A	T
26	TLCD1	17	28724748	Missense_Mutation	SNP	C	T
26	TLN1	9	35698441	Missense_Mutation	SNP	T	C
26	TM4SF5	17	4780800	Silent	SNP	G	A
26	TMEM132D	12	129074006	Missense_Mutation	SNP	C	T
26	TMEM143	19	48345196	Silent	SNP	G	A
26	TMEM259	19	1010692	Silent	SNP	A	G
26	TMEM273	10	49165787	Missense_Mutation	SNP	G	A
26	TMEM79	1	156285665	Missense_Mutation	SNP	G	A
26	TNC	9	115046625	Missense_Mutation	SNP	C	T
26	TNFRSF1A	12	6329607	Missense_Mutation	SNP	G	A
26	TNFRSF6B	20	63696914	Silent	SNP	C	T
26	TRAV36DV7	14	22227233	Missense_Mutation	SNP	A	G
26	TRIB1	8	125436150	Silent	SNP	C	T
26	TRIB3	20	391453	Missense_Mutation	SNP	G	A
26	TRIM29	11	120137584	Missense_Mutation	SNP	G	A
26	TRIM42	3	140688105	Missense_Mutation	SNP	G	A
26	TRIM66	11	8649822	Silent	SNP	C	T
26	TRMO	9	97910056	Missense_Mutation	SNP	C	T

26	TRPM6	9	74761731	Missense_Mutation	SNP	T	C
26	TRPM8	2	233996434	Silent	SNP	C	T
26	TSKU	11	76795719	Missense_Mutation	SNP	G	A
26	TSTD2	9	97625837	Missense_Mutation	SNP	G	T
26	TTC39A	1	51288232	Silent	SNP	G	A
26	TTC7A	2	47024330	Missense_Mutation	SNP	G	C
26	TTN	2	178535370	Missense_Mutation	SNP	C	T
26	TTN	2	178770097	Silent	SNP	G	A
26	TUBGCP6	22	50218225	Missense_Mutation	SNP	C	T
26	TXK	4	48086551	Missense_Mutation	SNP	G	A
26	UBE2R2	9	33900205	Missense_Mutation	SNP	C	T
26	UBE4B	1	10158381	Silent	SNP	T	C
26	UGT1A1	2	233760497	Silent	SNP	C	T
26	UHRF1	19	4929401	Silent	SNP	T	C
26	USP47	11	11922718	Splice_Region	SNP	A	G
26	USP6	17	5155513	Missense_Mutation	SNP	G	A
26	UTRN	6	144437663	Silent	SNP	A	G
26	VANGL2	1	160424115	Silent	SNP	A	G
26	VN1R2	19	53258522	Silent	SNP	C	T
26	VPS13A	9	77405974	Missense_Mutation	SNP	G	A
26	VPS13D	1	12373804	Silent	SNP	G	A
26	VWDE	7	12389177	Missense_Mutation	SNP	G	A
26	WDR19	4	39214601	Splice_Region	SNP	C	T
26	WDR27	6	169670627	Missense_Mutation	SNP	A	G
26	WDR33	2	127764843	Missense_Mutation	SNP	G	A
26	WDR7	18	56691281	Silent	SNP	C	T
26	WNT10B	12	48968256	Missense_Mutation	SNP	G	A
26	WNT5A	3	55470203	Silent	SNP	G	A
26	ZBTB38	3	141443286	Missense_Mutation	SNP	C	G
26	ZBTB38	3	141443343	Missense_Mutation	SNP	T	G
26	ZC3H18	16	88631105	Missense_Mutation	SNP	C	T
26	ZC3H4	19	47082260	Silent	SNP	G	C
26	ZNF169	9	94300699	Missense_Mutation	SNP	C	T
26	ZNF23	16	71449429	Missense_Mutation	SNP	G	A
26	ZNF236	18	76895162	Missense_Mutation	SNP	G	A
26	ZNF28	19	52800274	Missense_Mutation	SNP	A	G
26	ZNF28	19	52800452	Missense_Mutation	SNP	T	G

26	ZNF318	6	43339388	Missense_Mutation	SNP	C	T
26	ZNF341	20	33791457	Silent	SNP	T	C
26	ZNF346	5	177050911	Silent	SNP	G	A
26	ZNF35	3	44659088	Missense_Mutation	SNP	G	A
26	ZNF469	16	88435563	Missense_Mutation	SNP	T	A
26	ZNF519	18	14106040	Missense_Mutation	SNP	T	C
26	ZNF554	19	2834375	Silent	SNP	C	T
26	ZNF607	19	37698715	Silent	SNP	T	C
26	ZNF638	2	71427045	Missense_Mutation	SNP	G	A
26	ZNF684	1	40547054	Missense_Mutation	SNP	A	C
26	ZNF732	4	271236	Missense_Mutation	SNP	G	A
26	ZNF761	19	53456089	Missense_Mutation	SNP	G	A
26	ZNF793	19	37537551	Missense_Mutation	SNP	G	A
26	ZNF836	19	52157026	Missense_Mutation	SNP	C	T
26	ZNF836	19	52156738	Silent	SNP	A	G
26	ZNF860	3	31989643	Silent	SNP	A	G
26	ZPBP2	17	39868373	Silent	SNP	C	T
26	ZYG11B	1	52771524	Missense_Mutation	SNP	G	A
