# Supplementary material for: Association of genetic variants and survival in patients with acute myeloid leukemia in rural Appalachia
Source: Cancer Rep (Hoboken). 2022 Nov 16;6(3):e1746. doi: 10.1002/cnr2.1746 (PMC10026309; doi:10.1002/cnr2.1746)
Supplement: Supplementary file 2 — Figure S1: Mutational Landscape. Panel A shows oncoplot of mutated genes included in Mayo OncoHeme gene list compared to those of Panel B, the TCGA database. [file CNR2-6-e1746-s003.docx]

**SUPPLEMENTAL**

**
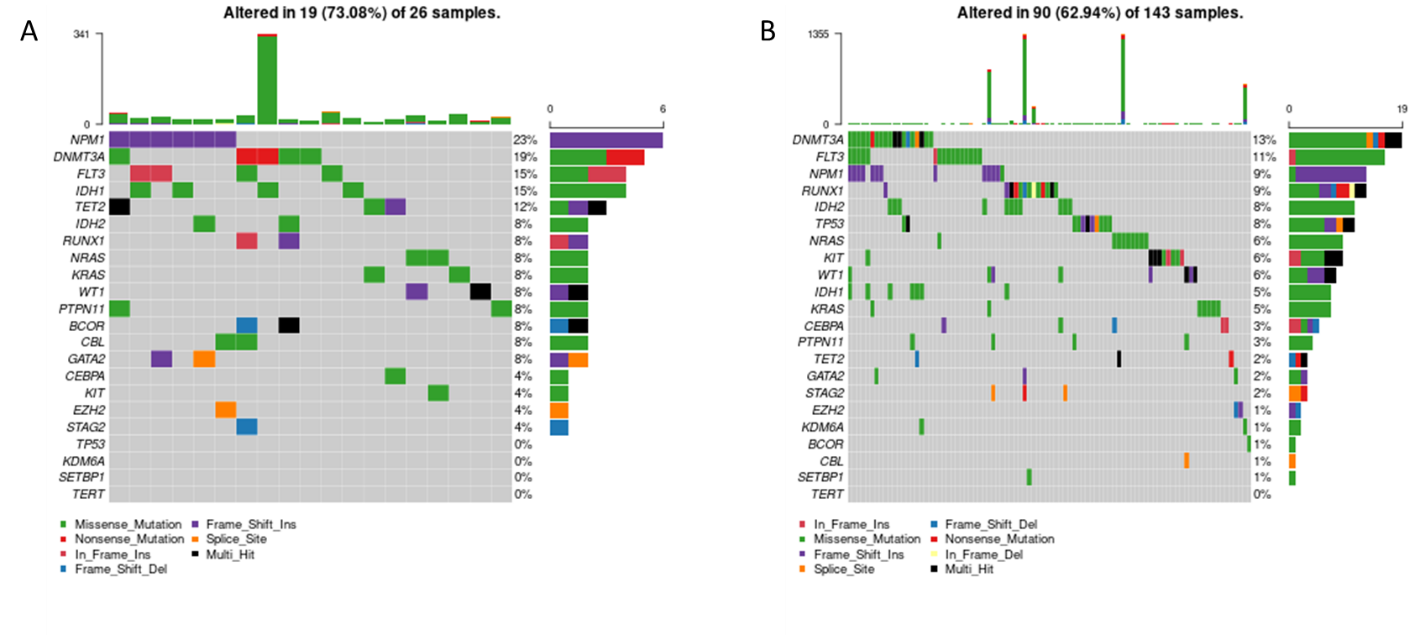

Figure 1:** Mutational Landscape. Panel A shows oncoplot of mutated genes included in Mayo OncoHeme gene list compared to those of Panel B, the TCGA database.
